# Highly Functionalized 1,2–Diamino Compounds through Reductive Amination of Amino Acid-Derived β–Keto Esters

**DOI:** 10.1371/journal.pone.0053231

**Published:** 2013-01-07

**Authors:** Paula Pérez-Faginas, M. Teresa Aranda, M. Teresa García-López, Lourdes Infantes, Asia Fernández-Carvajal, José Manuel González-Ros, Antonio Ferrer-Montiel, Rosario González-Muñiz

**Affiliations:** 1 Instituto de Química-Médica, IQM-CSIC, Madrid, Spain; 2 Instituto de Química Física Rocasolano IQFR-CSIC, Madrid, Spain; 3 Instituto de Biología Molecular y Celular, Universidad Miguel Hernández, Elche, Spain; University of Sydney, Australia

## Abstract

1,2-Diamine derivatives are valuable building blocks to heterocyclic compounds and important precursors of biologically relevant compounds. In this respect, amino acid-derived β–keto esters are a suitable starting point for the synthesis of β,γ–diamino ester derivatives through a two-step reductive amination procedure with either simple amines or α–amino esters. AcOH and NaBH_3_CN are the additive and reducing agents of choice. The stereoselectivity of the reaction is still an issue, due to the slow imine-enamine equilibria through which the reaction occurs, affording mixtures of diastereoisomers that can be chromatographically separated. Transformation of the β,γ–diamino esters into pyrrolidinone derivatives allows the configuration assignment of the linear compounds, and constitutes an example of their potential application in the generation of molecular diversity.

## Introduction

Reductive amination of carbonyl compounds is one of the most useful and versatile methods for the synthesis of different kinds of amines, key intermediates in organic synthesis and in the preparation of important building blocks for drug discovery [1]–[3]. Reductive amination proceeds upon reaction of a carbonyl compound with ammonia, a primary amine or a secondary amine, through the formation of a carbinolamine, which normally dehydrates to form an imine or an iminium ion intermediate, followed by *in situ* reduction to the corresponding amine alkylated product [2]. The process could be direct, when all components and reactives are mixed without prior formation of intermediates, or indirect, with pre-formation of intermediates (imine/iminium/enamine) and reduction in separate consecutive steps [3], [4]. Regarding the reduction process, the most used methods are catalytic hydrogenation and hydride agents [1]–[4], although some other reagents have been developed [5]–[7]. Reductive amination of aldehydes and ketones with primary amines are typically easy, fast, and high-yielding reactions with many examples documented in the literature [1]–[4]. However difficulties have been described for some aromatic and acyclic ketones, with slower reaction rates and lower isolated yields than those found for alicyclic ketones and aldehydes [4]. The rate of reaction also depends on the steric and electronic factors of the reactant amine, and the process usually requires the addition of AcOH, the use of 5–10% excess of the amine, and a large excess of the reducing agent [3], [4].

**Figure 1:**
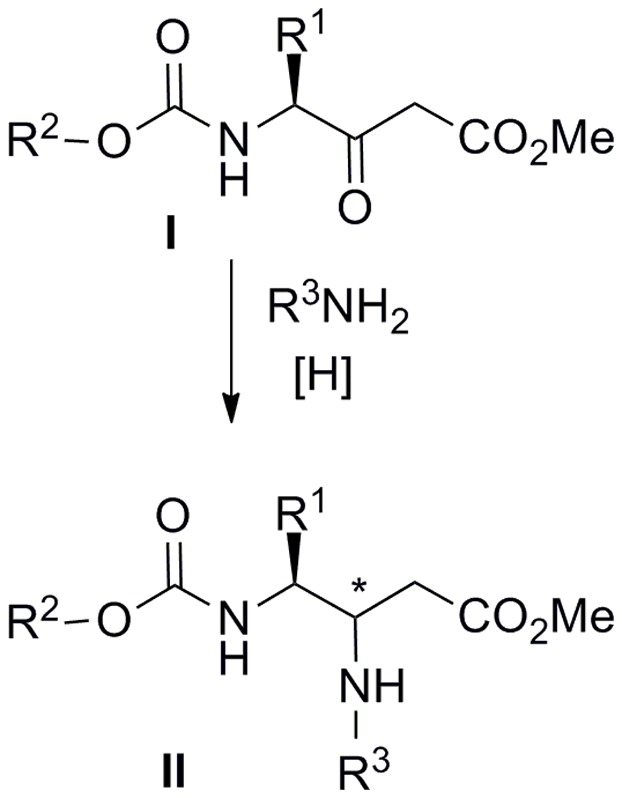
Intermolecular reductive amination of amino acid-derived β–ketoesters.

Examples of reductive amination using β-ketoesters as the carbonyl component are scarce, despite the final products, β–amino acid derivatives, have interesting synthetic and biological applications [8], [9]. A few reported examples describe the reduction of simple β–enamino esters by either catalytic hydrogenation or treatment with hydrides [10]–[12]. Other examples report the direct or indirect reductive amination of β–keto esters with ammonium acetate, different amines or the chiral ammonia equivalent α–methylbenzylamine [13]–[16]. In addition, both inter- and intramolecular processes have been applied to the efficient preparation of bioactive and natural compounds of high added value [17], [18]. Despite the well documented use of amino acids in the reductive amination of aldehydes (*i.e.* in the formation of peptide reduced bonds) [19], to the best of our knowledge, only two reports describe the application of amino acid derivatives with ketones and β–keto esters [20], [21]. In close relation to these precedents, we have previously studied the intramolecular reductive amination of Orn-derived β–keto esters (I, R^1^ = (CH_2_)_4_NH_2_) and some dipeptide analogues for the preparation of piperidine and piperazine heterocycles [22], [23]. These compounds were used as versatile chemical intermediates for the synthesis of highly substituted dioxoperhydropyrido[1,2-*c*]pyrimidine and trioxoperhydropyrazino[1,2-*f*]pyrimidine bicyclic systems [24], [25], the former successfully used as the central core of selective CCK1 receptor antagonists. Owing to this versatility, we decided to investigate the intermolecular version of this process starting from amino acid-derived β-ketoesters **I**. A suitable method for the reductive amination of compounds **I** could provide highly functionalized β,γ-diamino esters **II** (Figure 1), which can be seen as interesting intermediates for the generation of molecular diversity (*i.e.*, cyclization to different heterocyclic systems can easily be envisaged). Moreover, compounds **II** could bear additional reactive functions at R^1^ (starting amino acid side-chain) and amine R^3^ substituent, thus amplifying the possibilities of additional chemical manipulation. As the use of β-ketoesters in reductive amination processes is underdeveloped, many questions remain to be answered: Could amino acid-derived β-ketoesters **I** be applied to such an intermolecular process? Will the initial amino acid chiral center survive under the reductive amination conditions? Can amino acids be used as the amino component to incorporate additional complexity in final compounds? To answer these queries, we now describe our attempts to synthesize compounds **II**, through reductive amination, and the careful examination of the stereochemical issues.

## Results and Discussion

**Figure 2:**
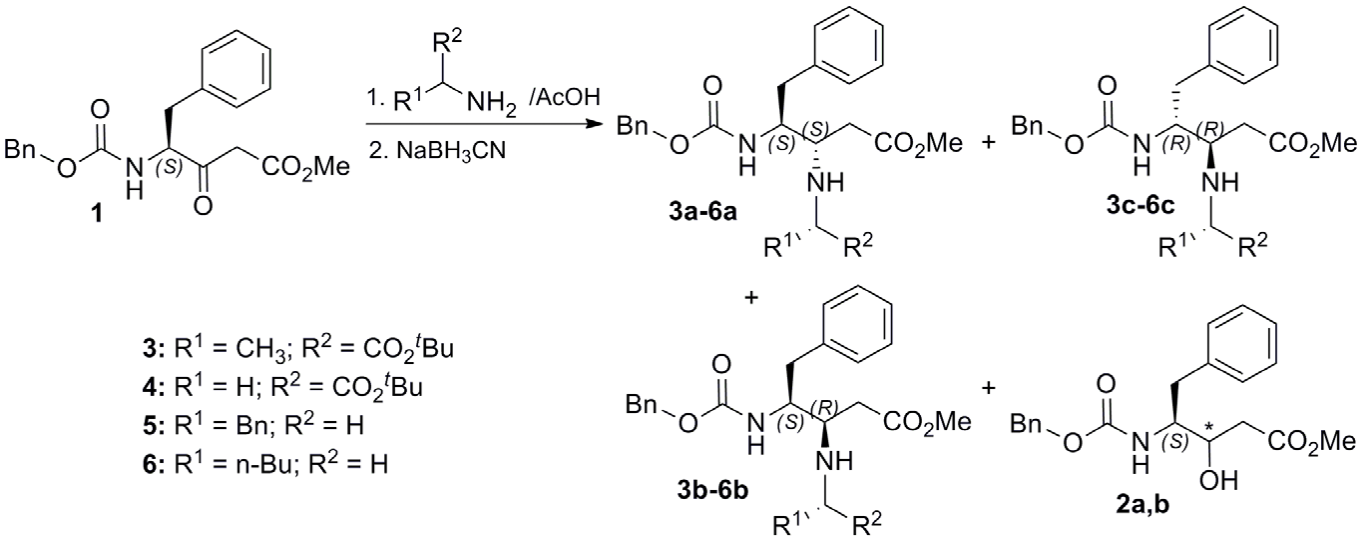
Synthetic procedure for β,γ–diamino esters by reductive amination of Phe-derived β–ketoester 1.

To explore the reaction between amino acid-derived β-ketoesters and amines we selected compound **1**, easily prepared from Z-Phe-OH following a previously described method from our lab [26]. Two primary amines (BnNH_2_ and *n*-BuNH_2_) and two α-amino esters (H-Ala-O*^t^*Bu and H-Gly-O*^t^*Bu) were chosen as amines for the reductive amination (Figure 2). H-Ala-O*^t^*Bu was selected for the initial exploratory study, since it could be the most demanding amino component. It is chiral, sterically congested due to α–substitution, and the lower pKa of its amino group (calculated pKa = 7.82) should normally imply a weaker nucleophilic character, and hence a lower reactivity than simple alkyl/benzyl amines (*n*-BuNH_2_, pKa = 10.87; BnNH_2_, pKa = 9.33).

Starting from **1** and H-Ala-O*^t^*Bu, we first investigated a battery of different conditions described for the reductive amination of ketones and β-keto esters, both using direct and indirect protocols. Direct reductive amination assays (Ti(O*^i^*Pr)_4_ or AcOH as additive and NaBH_3_CN or NaBH(OAc)_3_ as reducing agent failed, recovering the starting β–keto ester or leading to alcohols **2a,b** in different yield and diastereomeric ratio (Table S1, supporting information). Probably, the low reactivity of both carbonyl and amino species could take account for the disappointing results and, in fact, the diamino derivatives **3** were only detected, although in low yield, in the direct reaction at 50°C with AcOH/NaBH_3_CN. Using indirect procedures, first the reaction was allowed to stand at room temperature or at 50°C for the formation of the imine/enamine intermediates (completion monitored by tlc), in the presence of different additives commonly used in reductive aminations [Ti(O*^i^*Pr)_4_, AcOH, CAN, LaCl_3_, ZnCl_2_] [20], [27]–[30]. Then, NaBH_3_CN, NaBH(OAc)_3_, NaBH_4_, or H_2_/Pd-C were considered for the reduction of formed intermediates (Table S2, supporting information). The results of the two-step methods were more satisfactory, especially for the combination of AcOH and NaBH_3_CN, although the process required long reaction times, both for the intermediate formation and for the reduction step. Under the best conditions, (1. AcOH (1 equiv), CHCl_3_, 50°C; 2. NaBH_3_CN), the reaction of **1** and H-Ala-O*^t^*Bu afforded three diastereoisomeric compounds **3** (crude HPLC **a**:**b**:**c** ratio, 10∶13:17), which were chromatographically separated and their configuration established as indicated later on. Although it has been pointed out that the reductive amination of α–substituted β-keto esters apparently occurs with control of the stereochemistry at both α and β-positions [4], the formation of the 4*R*-configured isomer **3c** indicates that the stereochemical integrity at γ-position of the starting β–keto ester **1** was partially lost during the process. The reaction between **1** and H-Ala-O*^t^*Bu in the presence of Ti(O*^i^*Pr)_4_, followed by reduction with a mixture of NaBH(OAc)_3_ and NaBH_3_CN, afforded low yield of diastereoisomers **3a** and **3b**, along with alcohols **2a,b** as major products. In this case, the lack of **3c** suggests that most probably the intermediate species for reduction are hemiaminal titanate derivatives and not imine/enamine species [27]. In fact, the imine/enamine intermediates were detected by HPLC-MS in the Ti(O*^i^*Pr)_4_-promoted reactions in MeOH but not in the experiments performed in aprotic solvents (dichloromethane and dichloroethane, Table S2). Unfortunately, an attempt to optimize this Ti(O*^i^*Pr)_4_-mediated process, which included heating the mixture of the ketoester and the amine in neat Ti(O*^i^*Pr)_4_ (no solvent), was unsuccessful (Table S2, entry 8). The assay to reduce the preformed imine/enamine intermediates in the presence of a heterogeneous hydrogenation catalyst (Pd-C, 50°C, 45 psi), a method that have worked very well for other substrates [23], [31], was also unproductive in this case, resulting in the partial reversion to the initial β-ketoester (Table S2, entry 17).

Application of the AcOH/NaBH_3_CN optimized conditions to the reaction of **1** with H-Gly-O*^t^*Bu resulted in an approximately 1∶1 mixture of the expected diastereoisomers **4a** and **4b** (Figure 2, Table 1). Almost equimolecular mixtures of 3*S*,4*S* and 3*R*,4*S* diastereoisomers were also formed in the reaction with benzyl and butyl amines, although the total yield of the corresponding compounds **5a,b** and **6a,b** were slightly lower than those obtained with amino acids. Taking into account the higher pKa of these amines in relation to amino esters, this result seems to suggest that the amino acid-derived β–keto ester is the principal responsible of the low reactivity found. We might speculate that the existence of the ZNH group at the γ–position, neighboring to the reactive carbonyl, hampers the attack of the amine component. Finally, according to chiral HPLC experiments, compounds **4–6** were obtained as racemic mixtures, while a 70∶30 ratio of enantiomers was observed for Ala derivatives **3a–c** (Figures S1 and S2).

**Table 1:** Result of the reductive amination of Phe-derived β–ketoester **1**.

Final Compd.	Diastereoisomer (%)^a^		
	a	b	c
**3**	18	20	30
**4**	33	34	−
**5**	16	19	−
**6**	23	27	−

a Yield of isolated compounds.

**Figure 3:**
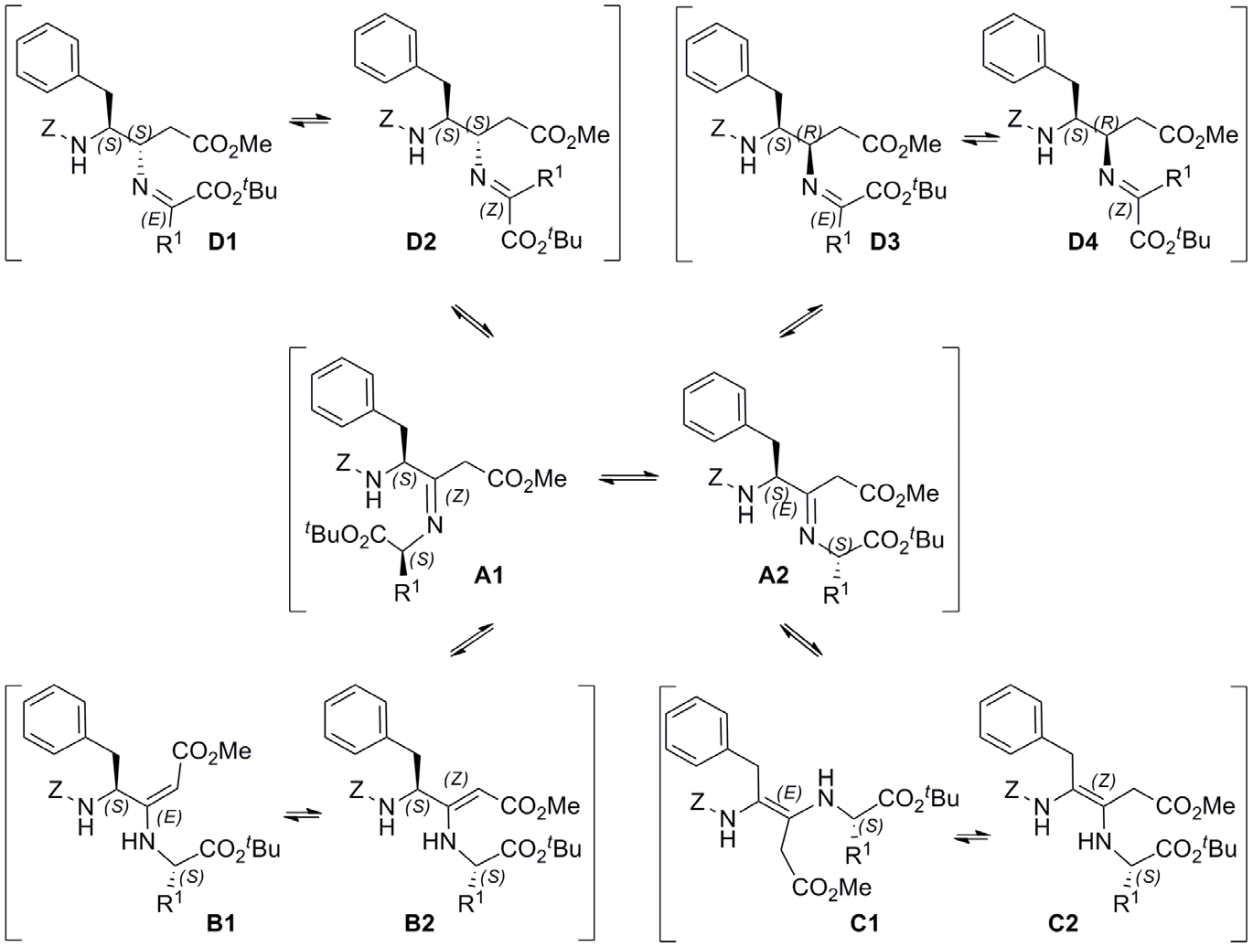
Reaction Intermediates. Possible intermediates in the reductive amination of **1** with H-Gly-O*^t^*Bu (R^1^ = H) or H-Ala-O*^t^*Bu (R^1^ = Me). For clarity, only 4*S* and 1′*S* isomers are depicted (**A–D**), but 4*R* and 1′*R* containing intermediates (**A’–D’**) are also possible if all the indicated species are present in equilibrium.

To provide some insight into the mechanism of the two-step reductive amination process, we follow first the formation of reaction intermediates by ^1^H NMR. The reaction of **1** and H-Gly-O*^t^*Bu in CDCl_3_ and AcOH (1 equiv) gave a mixture of *E* and *Z* enamines **B1** and **B2** in a 3∶4 ratio, as deduced from the singlet signals at 4.68 and 4.45 ppm, respectively [29] (Figure S3). However the spectrum of the crude reaction with H-Ala-O*^t^*Bu showed four signals of enamine proton (at 4.68, 4.62, 4.56 and 4.52 ppm, relative ratio 5∶8:9∶11), which were supposed to be two E- (**B1** and **B1’**) and two Z-isomers (**B2** and **B2’**), having 4*S* and 4*R*-configuration. From this result, we reasoned that the initially formed imines **A** should isomerize to the most stable conjugated enamines **B**, and that enamines **C** should also be present in the equilibrium and could be responsible for the stereochemical integrity loss (Figure 3). The observed epimerization at the 1′-position of final diamino esters indicated that conjugated-imines **D** must also be present among the possible intermediates of the reaction. While the imine-enamine tautomerism (A↔B and/or A↔C) was expected to occur in some extent, as previously reported for intramolecular processes [24], [32], the A↔D interconversion was unanticipated, since the chiral integrity of the amino acid derivative acting as the amino component is normally preserved in reductive amination reactions of α-amino esters with aldehydes, α-aminoaldehydes and even ketones [33]–[36].

When the intermediates formed between **1** and H-Ala-O*^t^*Bu or H-Gly-O*^t^*Bu were reduced with NaBD_3_CN the measured incorporation of deuterium (Table 2) corroborated the presence of intermediates **B**, **C** and **D**, although imines **A** are predominantly reduced by the hydride, as deduced from the high percentage of deuterium found at position 3 (Figures S4 and S5). The formation of all possible intermediates could be favored by the temperature and long times needed in the first step, due to the low reactivity of the starting materials, and to the slow speed of reduction in the second. Here, the more stable conjugated enamines **B** are reduced in low extent, but the transitory short-life imine **A** is the main intermediate trapped by the hydride.

**Table 2:** Incorporation of deuterium in the reduction with NaBD_3_CN.

Final Compd.	%D^a^			
	H2	H3	H4	H1’
**3a**	11	>90	5	4
**3b**	5	>90	3	6
**3c**	10	>90	5	3
**4a**	4	>90	4	5
**4b**	3	>90	3	5.5

a Measured by ^1^H NMR (d1 = 10) in CDCl_3_ at 25°C. Reduction of imines **A**: incorporation of D at H3; Enamines **B**: D at H2, H3; Enamines **C**: D at H3, H4; Imines **D**: D at H1’.

**Figure 4:**
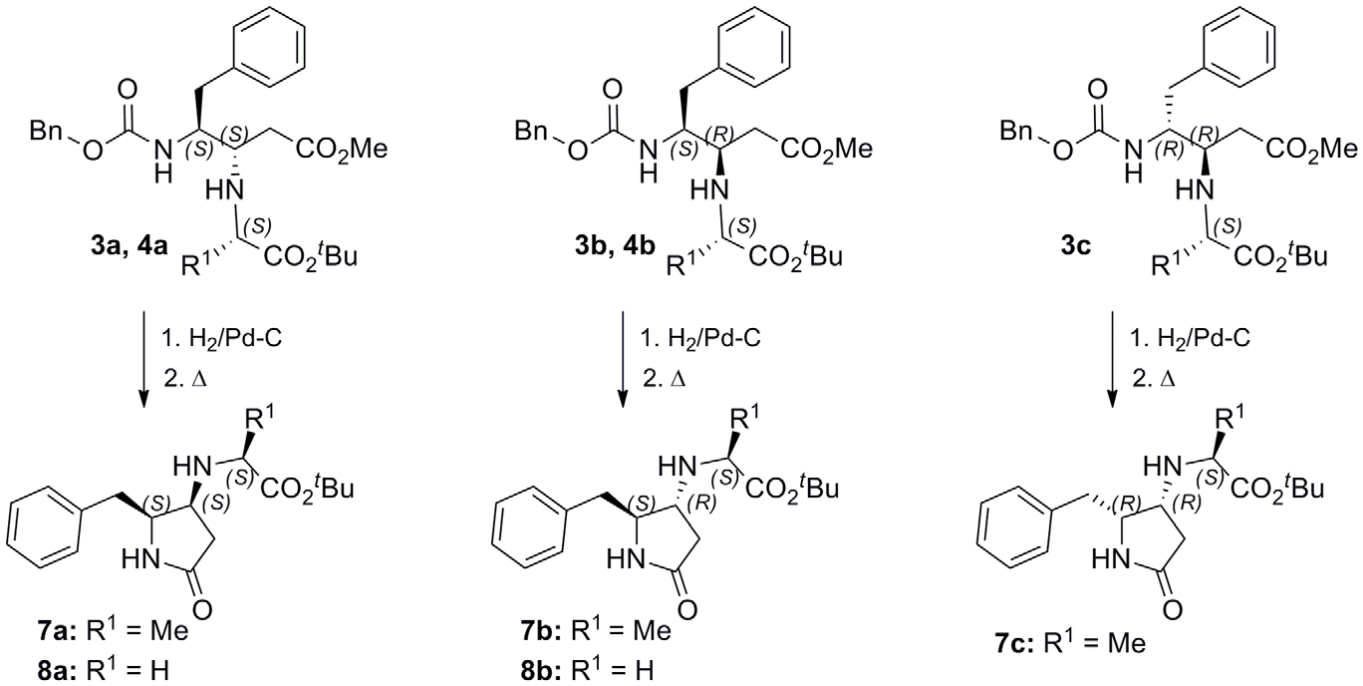
Synthetic procedure for 4,5-disubstituted 2-pyrrodinones from β,γ–diamino ester derivatives.

In the ^1^H NMR spectra, compounds **3a**, **3c** and **4a** showed a small value of the 3,4 coupling constant (0–2.9 Hz), indicating a preferred conformation in which the 3 and 4 protons form a dihedral angle close to 90°, while for isomers **b** this *J* value is higher (∼6.2 Hz). Although simple Chem3D calculation suggested a *threo* disposition for isomers **a** and **c** and *erythro* for **b**, this data did not afford any conclusive experimental information about the configuration at C3 and C4 chiral centers. The configurational assignment was done in an indirect way through the formation of pyrrolidinone derivatives. To this end, compounds **3** and **4** were deprotected at the 4-NH group and cyclized to the corresponding five-membered heterocycles **7** and **8**, respectively (Figure 4). These cyclic compounds can illustrate one example of the application of the described diamino esters in the creation of diverse heterocyclic scaffolds of interest. Related pyrrolidinone derivatives, having an unsubstituted 4-amino group, have been prepared through the Zinc-mediated homologation of α-aminonitriles and subsequent acidic hydrolysis [37]. The *J*_4,5_ in derivatives **7a**, **7c** and **8a** (∼6.5 Hz) was higher than in their corresponding distereoisomers **7b** and **8b** (∼4.5 ppm). Knowing that in this type of heterocyclic system the coupling constant are *J_anti_*<*J_syn_* [38], we can anticipate the *syn*- and *anti*-relative stereochemistry for isomers **a** (**c**) and **b**, respectively. Similarly, NOE experiments indicated a *syn*-relationship between H4 and H5 protons in **7a**, **7c** and **8a** and an *anti*-disposition in the respective isomers **b**. The exclusive formation of isomers **3a** and **3b** in a Ti(O*^i^*Pr)_4_ experiment, which probably occurs through titanate intermediates, allowed to distinguish between **3a** and **3c** *syn*-diastereoisomers. 4,5-*Syn*- and 4,5-*anti*-pyrrolidinones showed a different pattern of chemical shifts in ^13^C-NMR, with C-4, C-5, and especially 5-CH_2_ carbons notoriously shielded for *syn*-isomers **a** and **c** (∼55, 59, and 36 ppm) [39] with respect to their corresponding *anti* analogues **b** (57.5, 62, and 41 ppm). Just the opposite behavior was observed for the C-5 carbon in the linear precursors **3** and **4**, for which the most shielded signal corresponds to the *threo*-isomers **b** (∼37 ppm for *threo*, and ∼39 ppm for *erithro*). A comparison of these values with those of J*_3,4_* between distereoisomers **a** and **b** in compounds **5** and **6** allowed us the stereochemical assignment of these compounds.

**Figure 5:**
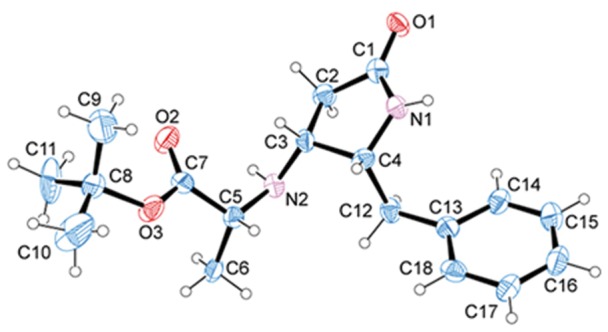
X-Ray molecular structure of 2-pyrrolidinone derivative 4R*,5R*–7a. Atom labeling for molecule 1 or 2 can be obtained by adding 100 or 200 respectively to the label of the atom shown in this figure, *i.e.* O1 is labelled O101 in molecule 1 and O201 in molecule 2. Thermal ellipsoids are drawn at 50% probability level of non-H atoms, and the H atoms are denoted as spheres of 0.1 Å radius.

**Figure 6:**
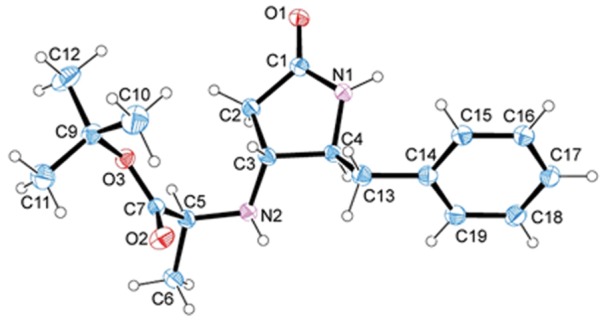
X-Ray molecular structure of 2-pyrrolidinone derivative 4R*,5S*–7b showing the atomic numbering scheme. Thermal ellipsoids are drawn at 50% probability level of non-H atoms, and the H atoms are denoted as spheres of 0.1 Å radius.

Fortunately, we succeed in getting crystal structures of a couple of the pyrrolidinone derivatives, **7a** and **7b**, corroborating the *syn* and *anti* disposition between substituents at 4 and 5 positions in these compound, and hence the previous assignment performed by NMR (Figures 5 and 6). Structures have been deposited at the Cambridge Crystallographic Data Centre, CCDC number: 880360 (**7a**) and 877464 (**7b**). Despite the 70∶30 enantiomeric mixture observed in chiral HPLC of these compounds, each crystal contains a racemic 1∶1 mixture of enantiomers. For **7a**, these enantiomers, related by a pseudocenter of symmetry [40], are forming dimers through N101-H101…O201 and N201-H201…O101 hydrogen bonds. Strong chains are created via a number of CH…π and CH…O = C contacts (C104-H104…Cen2, C103-H103…Cen2, C204-H204…Cen1, C203-H203…Cen1, C105-H105…O101, C205-H205…O201). These chains form (001) layers through CH…π interactions and the 3D structure is build up through CH…O = C contacts and Van der Waals interactions between the *tert*-butyl groups (Figure S6). For **7b**, the enantiomers, related by a center of symmetry, form dimers through N1-H1…O1 strong hydrogen bonds. These dimers are joined (N2-H2…O2) to form chains along the ac diagonal. These chains form (10-1) sheets through CH…O weak interactions (C10-H10a…O1 and C13-H132…O1). The crystal is formed by the union of these sheets through CH…π (C12-H12A…CenPh) contacts (Figure S7).

### Conclusions

In summary, we describe a procedure for the preparation of β,γ–diamino esters from reaction of amino acid-derived β–ketoesters with both simple amines as well as α–amino esters. To the best of our knowledge, this represents the first reductive amination protocol ever described using amino acid-derived β–ketoesters. This requires a first step of formation of intermediates with AcOH, and the subsequent reduction with NaBH_3_CN. In this method, the diastero- and enantioselectivities were compromised by the existence of different imine-enamine equilibria, as demonstrated by ^1^H NMR and deuteration experiments. These equilibria are favored by the long reaction times required, probably derived from the low reactivity of the hindered carbonyl component. The separated diastereoisomeric β,γ–diamino esters can be transformed into the corresponding pyrrolidinone derivatives by cyclization between the 4-amino and the 1-carboxylate groups. These pyrrolidinones serve as reliable clue in the configurational assignment (NMR, X-ray) of the linear precursors, and represent a first example of the potential of the described diamino esters for the preparation of different heterocycles.

## Materials and Methods

All reagents were of commercial quality. Solvents were dried and purified according to standard methods. Flash chromatography was performed on silica gel 60 (230–400 mesh). NMR spectra were recorded on spectrometers operating at 300 and 75 MHz for ^1^H and ^13^C, respectively, using TMS as internal standard (Figure S8). Chemical shifts are given in ppm and *J* values in Hz. The C attributions are supported by HSQC experiments. Electrospray mass spectra (positive mode) were also recorded. Analytical HPLC were performed on a Eclipse Plus C_18_ (5 µm, 4.6×150 mm) column using a UV detector at 220 nm. Mixtures of CH_3_CN (0.05% TFA, solvent A) and H_2_O (solvent B) were used in the mobile phase, and the corresponding mixture was specified in each case (flow rate 1 mL/min). HPLC-MS, performed in a X-Bridge C_18_ (3.5 µm, 2.1×100 mm) column; eluent CH_3_CN (0.08% formic acid, solvent A) and H_2_O (0.1% formic acid, solvent B); flow rate 0.5 mL/min. Chiral HPLC was performed on a Chiralpak IA column (0.4×25 cm) using the mixtures of solvents indicated in each case (isocratic conditions). β–Ketoester **1** was prepared as previously described [41], [42].

### General Procedure for the Reductive Amination

Method A (One step-procedure). To a stirred solution of β-ketoester **1** (57 mg, 0.15 mmol) in the appropriate solvent (2 mL), H-Ala-O^t^Bu (0.30 mmol), TEA (41 µL, 0.30 mmol) the corresponding additive (0.5–2 equiv.) and the reducing agent (20 mg, 0.30 mmol) were added. The mixture was stirred at room temperature or 50°C for 1–3 days. After evaporation, the residue was dissolved in EtOAc and washed with 10% NaHCO_3_ and brine, dried over Na_2_SO_4_ and concentrated in vacuum. The resulting residue was monitored by HPLC-MS (Table S1), and in one case purified on a silica gel column using hexane:EtOAc 4∶1 to characterize alcohol derivatives **2**.

### (3*R*,4*S*)-Methyl 4-(benzyloxycarbonyl)amino-3-hydroxy-5-phenylpentanoate (2a)

HPLC *t*_R_ = 10.78 min (X-Bridge, gradient 20–100 ACN in 15 min.). ^1^H NMR (CDCl_3_) δ: 7.44–7.12 (m, 10H, Ar), 5.22 (bd, 1H, *J* = 7.4, N*H* Z), 5.11 and 5.06 (d, 1H, *J = *12.4 Hz, C*H*_2_ Z ), 4.01 (bd, 1H, *J* = 9.6, 3-*H*), 3.82 (q, 1H, *J* = 7.9, 4-*H*), 3.67 (s, 3H, OC*H*_3_), 3.42 (bs, 1H, OH), 2.94 (bd, 2H, *J* = 7.6, 2-*H*), 2.59 (dd, 1H, *J* = 16.9, 10.2, 5-*H*), 2.39 (dd, 1H, *J* = 16.9, 2.3, 5-*H*). ^13^C NMR (CDCl_3_) δ:173.8 (*C*O_2_), 156.3 (*C*O Z), 137.9, 136.5 (*C* Ar), 129.4, 128.6, 128.5, 128.1, 127.9, 126.5 (*C*H Ar), 66.9 (3-*C*), 66.8 (*C*H_2_ Z), 55.9 (4-*C*), 51.9 (O*C*H_3_), 38.6 (2-*C*), 38.3 (5-*C*). [α]_D_ = +12.5 (*c*, 0.8 MeOH).

### (3*S*,4*S*)-Methyl 4-(benzyloxycarbonyl)amino-3-hydroxy-5-phenylpentanoate (2b)

Mp: 123–126°C. HPLC *t*_R_ = 10.5 min (X-Bridge, gradient 20–100 ACN in 15 min.). ^1^H NMR (CDCl_3_) δ: 7.46–7.10 (m, 10H, Ar), 5.02 (s, 2H, C*H*_2_ Z) 4.82 (m, 1H, N*H* Z), 4.03 (m, 1H, 3-*H*), 3.95 (m, 1H, 4-*H*), 3.71 (s, 3H, OC*H*_3_), 3.48 (bs, 1H, OH), 3.00 (dd, 1H, *J* = 14.1, 4.5, 5-*H*), 2.86 (m, 1H, 5-*H*), 2.60 (dd, 1H, *J* = 16.6, 3.5, 2-*H*), 2.52 (dd, 1H, *J* = 16.6, 8.6, 2-*H*). ^13^C NMR (CDCl_3_) δ:173.2 (*C*O_2_), 156.1 (*C*O Z), 137.3, 136.3 (*C* Ar), 129.4, 128.5, 128.4, 128.0, 127.9, 126.5 (*C*H Ar), 69.9 (3-*C*), 66.7 (*C*H_2_ Z), 55.8 (4-*C*), 51.9 (O*C*H_3_), 38.0 (5-*C*), 35.6 (2-*C*). [α] =  –40.0 (*c*, 0.5 MeOH). Described: [α]_D_ =  –45.5 (*c*, 1 MeOH) [43].

Method B (Two step-procedure, AcOH). To a stirred solution of β-ketoester **1** (0.57 g, 1.6 mmol) in CHCl_3_ (10 mL), the corresponding amine (4.8 mmol) and AcOH (91 µL, 1.6 mmol) were added. In the case of amino acid derivatives, the amino group was released from the HCl salt by addition of TEA (0.66 mL, 4.8 mmol). The mixture was stirred at 50°C until the total formation of imine/enamine intermediates was observed. Then, NaBH_3_CN (0.2 g, 3.2 mmol) was added, and the mixture was stirred at room temperature until complete reduction of the imine/enamine intermediates. After evaporation, the residue was dissolved in EtOAc and washed with 10% NaHCO_3_ and brine, dried over Na_2_SO_4_ and concentrated in vacuum. The resulting residue was purified on a silica gel column using the solvent system indicated in each case.

Method C (Two step-procedure, Ti(O^i^Pr)_4_). To a stirred solution of β-ketoester **1** (0.1 g, 0.28 mmol) in CH_2_Cl_2_ (3 mL), was added a solution of H-Ala-O*^t^*Bu.HCl (0.218 mmol) and TEA (0.218 mmol) in CH_2_Cl_2_ (3 mL). The mixture was stirred at room temperature overnight. Then the reaction was cooled to 0°C and NaBH_3_CN (0.563 mmol) and NaBH(OAc)_3_ (0.563 mmol) were added. The reaction was stirred overnight. After evaporation, the residue was solved in EtOAc and was washed with H_2_O and brine, dried over Na_2_SO_4_ and concentrated in vacuum. The resulting residue was purified on a silica gel column using EtOAc:hexane (1∶8 to 1∶4) as eluents. Alcohols **2a** (47.3 mg) and **2b** (27.2 mg) were isolated along with compounds **3a** (8.8 mg) and **3b** (5.6 mg).

#### Methyl 4-(benzyloxycarbonyl)amino-3-[1′-(*tert*-butoxycarbonyl)ethan-1′-yl]amino-5-phenylpentanoate (3). Diastereomer 3a (3S*,4S*,1′S*)

(CH_2_Cl_2_:Et_2_O:hexane, 1∶1:2). Yield: 18% (syrup). HPLC *t*_R_ = 13.66 min (5 to 100% A in 15 min). ^1^H NMR (CDCl_3_) δ: 7.37–7.10 (m, 10H, Ar), 5.15 (d, 1H, *J* = 9.4 Hz, 4-N*H*), 5.05, 4.98 (AB system, 2H, *J* = 12.2 Hz, C*H*_2_ Z), 3.96 (q, 1H, *J* = 7.6 Hz, 4-*H*), 3.61 (s, 3H, OC*H*_3_), 3.29 (q, 1H, *J* = 7.0 Hz, 1′-*H*), 2.99 (dd, 1H, *J* = 6.9, 5.7 Hz, 3-*H*), 2.84 (d, 2H, *J* = 7.6 Hz, 5-*H*), 2.51 (dd, 2H, *J* = 15.2, 6.9 Hz, 2-*H*), 2.44 (dd, 2H, *J* = 15.2, 5.7 Hz, 2-*H*),1.73 (bs, 1H, 3-N*H*), 1.44 (s, 9H, C*H*_3_ *^t^*Bu), 1.24 (d, 3H, *J* = 7.0 Hz, 2′-*H*).^ 13^C NMR (CDCl_3_) δ: 175.3, 172.0 (*C*O_2_), 156.1 (*C*O Z), 138.0, 136.6 (*C* Ar), 129.1, 128.4, 128.3, 127.9, 126.3 (*C*H Ar), 81.2 (*C ^t^*Bu), 66.5 (*C*H_2_ Z), 57.2 (3-*C*), 55.6 (4-*C*), 54.8 (1′-C), 51.6 (OMe), 39.1 (5-*C*), 38.1 (2-*C*), 27.9 (*C*H_3_ *^t^*Bu), 20.0 (2′-*C*). MS: 485.5 [M+1]^+^. Anal. cal. for C_27_H_36_N_2_O_6_: C 66.92, H 7.49, N 5.78, found C 66.88, H 7.51, N 5.65.

#### Diastereomer 3b (3R*,4S*,1′S*)

(CH_2_Cl_2_:Et_2_O:hexane, 1∶1:2). Yield: 20% (syrup). HPLC t_R_ = 12.55 min (5 to 100% A in 15 min).^1^H NMR (CDCl_3_) δ:7.32-7.19 (m, 10H, Ar), 5.34 (d, 1H, *J* = 7.8 Hz, 4-N*H*), 5.02 and 4.97 (AB system, 2H, *J* = 12.6 Hz, C*H*_2_ Z), 3.99 (m, 1H, 4-*H*), 3.65 (s, 3H, OC*H*_3_), 3.32 (q, 1H, *J* = 6.8 Hz, 1′-*H*), 3.13 (q, 1H, *J* = 6.3 Hz, 3-*H*), 2.94 (dd, 1H, *J* = 13.8, 5.3 Hz, 5-*H*), 2.78 (dd, 1H, *J* = 13.8, 8.8 Hz, 5-*H*), 2.51 (dd, 1H, *J* = 15.3, 5.8 Hz, 2-*H*), 2.78 (dd, 1H, *J* = 15.3, 6.9 Hz, 2-*H*), 1.68 (bs, 1H, 3-N*H*), 1.43 (s, 9H, C*H*_3_ *^t^*Bu),1.21 (d, 3H, *J* = 6.8 Hz, 2′-*H*).^13^C NMR (CDCl_3_) δ: 174.9, 172.5 (CO_2_), 156.1 (CO Z), 137.9, 136.7(C Ar), 129.2, 128.4, 128.3, 127.8, 127.7, 126.4 (CH Ar), 81.1 (*C*(CH_3_)_3_), 66.4 (*C*H_2_ Z), 56.8 (3-C), 55.4 (1′-C and 4-*C*), 51.7 (O*C*H_3_), 37.15 (5-*C*), 37.1 (2-*C*), 27.9 (C(*C*H_3_)_3_), 19.6 (2′-*C*). MS: 485.5 [M+1]^+^. Anal. cal. for C_27_H_36_N_2_O_6_: C 66.92, H 7.49, N 5.78, found C 66.67, H 7.83, N 5.72.

#### Diastereomer 3c (3S*,4S*,1′R*)

(CH_2_Cl_2_:Et_2_O:hexane, 1∶1:2). Yield: 30% (syrup). HPLC t_R_ = 12.90 min (5 to 100% A in 15 min).^1^H NMR (CDCl_3_) δ:7.32-7.19 (m, 10H, Ar), 5.35 (d, 1H, *J* = 8.7 Hz, 4-N*H*), 5.07 and 5.00 (AB system, 2H, *J* = 12.3 Hz, C*H*_2_ Z), 3.95 (m, 1H, 4-*H*), 3.61 (s, 3H, OC*H*_3_), 3.27 (q, 1H, *J* = 6.9 Hz, 1′-*H*), 3.12 (dt, 1H, *J* = 6.3, 2.9 Hz, 3-*H*), 2.93 (dd, 1H, *J* = 13.7, 6.3 Hz, 5-*H*), 2.81 (dd, 1H, *J* = 13.7, 7.7 Hz, 5-*H*), 2.48 (dd, 1H, *J* = 15.1, 6.7 Hz, 2-*H*), 2.40 (dd, 1H, *J* = 15.1, 6.4 Hz, 2-*H*), 1.73 (bs, 1H, 3-N*H*), 1.45 (s, 9H, C*H*_3_ *^t^*Bu), 1.23 (d, 3H, *J* = 6.9 Hz, 2′-*H*).^13^C NMR (CDCl_3_) δ: 174.9, 172.3 (CO_2_), 156.1 (CO Z), 137.8, 136.6 (C Ar), 129.3, 129.1, 128.4, 128.3, 127.9, 127.8, 126.3 (CH Ar), 81.2 (*C*(CH_3_)_3_), 66.5 (*C*H_2_ Z), 55.5 (4-*C*), 55.1 (1′-*C*), 54.4 (3-*C*), 51.5 (O*C*H_3_), 38.3 (5-*C*), 37.3 (2-*C*), 27.9 (C(*C*H_3_)_3_), 19.7 (2′-*C*). MS: 485.5 [M+1]^+^. Anal. cal. for C_27_H_36_N_2_O_6_: C 66.92, H 7.49, N 5.78, found C 66.90, H 7.35, N 5.51.

#### Methyl 4-(benzyloxycarbonyl)amino-3-[(*tert*-butoxycarbonyl)methyl]amino-5-phenylpentanoate (4). Diastereomer 4a (3S*,4S*)

(CH_2_Cl_2_:Et_2_O:hexane, 1∶1:3 to 1∶1:1). Yield: 33% (syrup). HPLC *t*_R_ = 12.33 min (20 to 100% A in 20 min). ^1^H NMR (CDCl_3_) δ: 7.35–7.21 (m, 10H, Ar), 5.20 (bd, 1H, *J* = 9.1 Hz, 4-N*H*), 5.06 and 4.99 (AB system, 2H, *J* = 12.4 Hz, C*H*_2_ Z), 3.97 (m, 1H, 4-*H*), 3.63 (s, 3H, OC*H*_3_), 3.36 (s, 2H, C*H*_2_ Gly), 3.03 (dt, 1H, *J* = 6.7, 2.2 Hz, 3-*H*), 2.87 (dq, 2H, *J* = 13.7, 7.4 Hz, 5-*H*), 2.47 (m, 2H, 2-*H*), 1.60 (bs, 1H, 3-N*H*), 1.46 (s, 9H, C*H*_3_ *^t^*Bu). ^13^C NMR (CDCl_3_) δ: 174.4 and 173.5 (*C*O_2_), 157.9 (*C*O Z), 139.6, 137.9, 136.6, 129.2, 128.4, 127.9, 127.8, 126.4 (*C* and *C*H Ar), 81.5 (C *^t^*Bu), 66.5 (*C*H_2_ Z), 56.1 (3-*C*), 55.6 (4-*C*), 51.7 (O*C*H_3_), 51.0 (*C*H_2_ Gly), 38.7 (5-*C*), 37.6 (2-*C*), 28.0 (*C*H_3_ *^t^*Bu). MS: 471.3 [M+1]^+^, 493.3 [M+23]^+^. Anal. cal. for C_26_H_34_N_2_O_6_: C 66.36, H 7.28, N 5.95, found C 66.55, H 7.14, N 5.75.

#### Diastereomer 4b (3R*,4S*)

(CH_2_Cl_2_:Et_2_O:hexane, 1∶1:3 to 1.1∶1). Yield: 34% (solid). HPLC *t*_R_ = 12.52 min (20 to 100% A in 20 min). ^1^H NMR (CDCl_3_) δ: 7.33–7.18 (m, 10H, Ar), 5.15 (d, 1H, *J* = 9.2 Hz, 4-N*H*), 5.00 (m, 2H, C*H*_2_ Z), 4.01 (m, 1H, 4-*H*), 3.66 (s, 3H, OC*H*_3_), 3.38 and 3.22 (AB system, 2H, *J* = 17.4 Hz, C*H*_2_ Gly), 3.09 (q, 1H, *J* = 6.1 Hz, 3-*H*), 2.93 (dd, 1H, *J* = 13.9, 5.1 Hz, 5-*H*), 2.79 (m, 1H, 5-*H*_2_), 2.50 (m, 2H, 2-*H*), 1.45 (s, 9H, C*H*_3_ *^t^*Bu). ^13^C NMR (CDCl_3_) δ: 172.5 and 171.7 (*C*O_2_), 156.0 (*C*O Z), 137.8, 136.6, 129.2, 128.4, 128.3, 127.9, 127.8, 126.4 (*C* and *C*H Ar), 81.3 (*C ^t^*Bu), 66.4 (*C*H_2_ Z), 57.8 (3-*C*), 55.0 (4-*C*), 51.8 (O*C*H_3_), 49.9 (*C*H_2_ Gly), 37.1 (5-*C*), 36.6 (2-*C*), 28.0 (*C*H_3_ *^t^*Bu). MS: 471.3 [M+1]^+^, 493.3 [M+23]^+^. Anal. cal. for C_26_H_34_N_2_O_6_: C 66.36, H 7.28, N 5.95, found C 66.58, H 7.39, N 5.62.

#### Methyl 3-benzylamino-4-(benzyloxycarbonyl)amino-5-phenylpentanoate (5). Diastereomer 5a (3S*,4S*)

(CH_2_Cl_2_:Et_2_O:hexane, 1∶1:2). Yield: 16% (syrup). HPLC *t*_R_ = 8.02 min (20 to 100% A in 20 min). ^1^H NMR (CDCl_3_) δ: 7.37–7.11 (m, 15H, Ar), 5.20 (bs, 1H, 4-N*H*), 5.05 and 5.00 (AB system, 2H, *J* = 12.0 Hz, C*H*_2_ Z), 4.01 (m, 1H, 4-*H*), 3.87 and 3.72 (AB system, 2H, *J* = 12.7 Hz, C*H*_2_ *N-*Bn), 3.62 (s, 3H, OC*H*_3_), 3.11 (m, 1H, 3-*H*), 2.87 (m, 2H, 5-*H*), 2.49 (m, 2H, 2-*H*), 1.60 (bs, 1H, 3-N*H*). ^13^C NMR (CDCl_3_) δ: 172.3 (*C*O_2_), 156.2 (*C*O Z), 137.9, 136.4, 129.1, 128.5, 128.4, 128.0, 127.9, 127.4, 126.4 (*C* and *C*H Ar), 66.6 (*C*H_2_ Z), 55.9 (4-*C*), 55.6 (3-*C*), 52.7 (*C*H_2_ Bn), 51.7 (O*C*H_3_), 38.8 (5-*C*), 31.7 (2-*C*). MS: 447.3 [M+1]^+^. Anal. cal. for C_27_H_30_N_2_O_4_: C 72.62, H 6.77, N 6.27, found C 72.29, H 6.82, N 5.93.

#### Diastereomer 5b (3R*,4S*)

(CH_2_Cl_2_:Et_2_O:hexane, 1∶1:2). Yield: 19% (solid). HPLC *t*_R_ = 8.03 min (20 to 100% A in 20 min). ^1^H NMR (CDCl_3_) δ:7.34–7.15 (m, 15H, Ar), 5.03 (m, 2H, C*H*_2_ Z), 4.92 (d, 1H, *J* = 9.3 Hz, 4-N*H*), 4.11 (m, 1H, 4-*H*), 3.81 (s, 2H, C*H*_2_ *N-*Bn), 3.64 (s, 3H, OC*H*_3_), 3.11 (m, 1H, 3-*H*), 2.94 (dd, 1H, *J* = 13.9, 5.2 Hz, 5-*H*), 2.78 (m, 1H, 5-*H*), 2.54 (m, 2H, 2-*H*). ^13^C NMR (CDCl_3_) δ: 172.7 (*C*O_2_), 156.2 (*C*O Z), 140.0, 137.8, 136.5, 129.2, 128.5, 128.4, 128.3, 128.2, 128.0, 127.9, 127.0, 126.5 (*C* and *C*H Ar), 66.6 (*C*H_2_ Z), 56.8 (3-*C*), 54.5 (4-*C*), 51.8 (O*C*H_3_), 51.2 (*C*H_2_ *N-*Bn), 37.7 (5-*C*), 35.9 (2-*C*). MS: 447.3 [M+1]^+^, 469.2 [M+23]^+^. Anal. cal. for C_27_H_30_N_2_O_4_: C 72.62, H 6.77, N 6.27, found C 72.47, H 6.95, N 6.05.

#### Methyl 4-(benzyloxycarbonyl)amino-3-butylamino-5-phenylpentanoate (6). Diastereomer 6a (3S*,4S*)

(CH_2_Cl_2_:Et_2_O:hexane, 1∶1:2). Yield: 23% (syrup). HPLC *t*_R_ = 7.52 min (20 to 100% A in 20 min). ^1^H NMR (CDCl_3_) δ: 7.35-7.19 (m, 10H, Ar), 5.12 (d, 1H, *J* = 8.0 Hz, 4-N*H*), 5.06 and 5.00 (AB system, 2H, *J* = 12.0 Hz, C*H*_2_ Z), 3.96 (m, 1H, 4-*H*), 3.63 (s, 3H, OC*H*_3_), 3.02 (m, 1H, 3-*H*), 2.87 (m, 2H, 5-*H*), 2.69 (m, 1H, 4-C*H*_2_ Bu), 2.54 (dt, 1H, *J* = 11.1, 5.2 Hz, 4-C*H*_2_ Bu), 2.44 (m, 2H, 2-*H*), 1.44-1.26 (m, 4H, 2- and 3-C*H*_2_ Bu), 1.20 (bs, 1H, 3-N*H*), 0.91 (t, 3H, *J* = 7.1 Hz, C*H*_3_ Bu). ^13^C NMR (CDCl_3_) δ: 172.5 (*C*O_2_), 156.2 (*C*O Z), 138.1, 136.6, 129.2, 128.5, 128.4, 128.0, 127.9, 126.4 (*C* and *C*H Ar), 66.6 (*C*H_2_ Z), 56.1 (3-*C*), 55.6 (4-*C*), 51.7 (O*C*H_3_), 48.5 (4-*C*H_2_ Bu), 38.8 (5-*C*), 37.3 (2-*C*), 32.7 (3-*C*H_2_ Bu), 20.3 (2-*C*H_2_ Bu), 13.9 (*C*H_3_ Bu). MS: 413.3 [M+1]^+^. Anal. cal. for C_24_H_32_N_2_O_4_: C 69.88, H 7.82, N 6.79, found C 70.18, H 7.61, N 6.80.

#### Diastereomer 6b (3R*,4S*)

(CH_2_Cl_2_:Et_2_O:hexane, 1∶1:3). Yield: 27% (syrup). HPLC *t*_R_ = 7.72 min (20 to 100% A in 20 min). ^1^H NMR (CDCl_3_) δ: 7.34-7.17 (m, 10H, Ar), 5.01 (m, 2H, C*H*_2_ Z), 4.92 (d, 1H, *J* = 9.2 Hz, 4-N*H*), 4.02 (m, 1H, 4-*H*), 3.65 (s, 3H, OC*H*_3_), 3.03 (q, 1H, *J* = 6.2 Hz, 3-*H*), 2.94 (dd, 1H, *J* = 13.8, 5.2 Hz, 5-*H*), 2.79 (m, 1H, 5-*H*), 2.59 (m, 2H, 4-C*H*_2_ Bu), 2.50 (m, 2H, 2-*H*), 1.44-1.26 (m, 5H, 3-N*H*, 2- and 3-C*H*_2_ Bu), 0.89 (t, 3H, *J* = 7.1 Hz, C*H*_3_ Bu). ^13^C NMR (CDCl_3_) δ: 172.9 (*C*O_2_), 156.1 (*C*O Z), 137.9, 136.6, 129.0, 128.5, 128.4, 128.0, 127.9, 126.4 (*C* Ar), 66.6 (*C*H_2_(Z)), 57.6 (3-*C*), 54.7 (4-*C*), 51.7 (O*C*H_3_), 46.8 (4-*C*H_2_ Bu), 37.6 (5-*C*), 36.1 (2-*C*), 32.5 (3-*C*H_2_ Bu), 20.3 (2-*C*H_2_ Bu), 13.9 (*C*H_3_ Bu). MS: 413.3 [M+1]^+^. Anal. cal. for C_24_H_32_N_2_O_4_: C 69.88, H 7.82, N 6.79, found C 69.98, H 7.93, N 6.55.

### General Procedure for the Cyclization to 2-pyrrolidinones

Diamino ester derivatives (0.2 mmol) were dissolved in MeOH (10 mL) and a catalytic amount of Pd/C (10% w/w) and HCl (35%) (0.2 mmol) were added. The reaction was kept under 20 psi of H_2_ for 2 h at room temperature. The suspension was filtered and evaporated. Then, the residue was dissolved in toluene (5 mL) and TEA (28 µL, 0.2 mmol) was added. The reaction was heated at 110°C for 2 hours. After evaporation, the residue was dissolved in EtOAc and washed with H_2_O and brine, dried over Na_2_SO_4_ and concentrated in vacuum. The resulting residue was purified on a silica gel column using the solvents indicated in each case.

### (4S*,5S*,1′S*)-5-Benzyl-4-[(1′-(*tert*-butoxycarbonyl)ethyl]amino-2-pyrrolidinone (7a)

Eluent: CH_2_Cl_2_:MeOH (50∶1). 46% (oil). HPLC *t*_R_ = 6.24 min (20 to 100% A in 20 min).^1^H NMR (CDCl_3_) δ:7.33–7.17 (m, 5H, H Ar), 5.46 (bs, 1H, 1-N*H*), 3.81 (dddd, 1H, *J* = 11.1, 6.7, 3.4, 0.7 Hz, 5-*H*), 3.56 (m, 1H, *J* = 9.3, 7.8, 6.7 Hz, 4-*H*), 3.22 (q, 1H, *J* = 7.0 Hz, 1′-*H*), 2.98 (dd, 1H, *J* = 13.5, 3.4 Hz, 5-C*H*_2_), 2.58 (dd, 1H, *J* = 13.5, 11.1 Hz, 5-C*H*_2_), 2.47 (dd, 1H, *J* = 16.4, 7.8 Hz, 3-*H*), 2.28 (dd, 1H, *J* = 16.4, 9.3 Hz, 3-*H*), 1.81 (bs, 1H, 4-N*H*), 1.48 (s, 9H, CH_3_ *^t^*Bu), 1.29 (d, 3H, *J* = 7.0, 2′-*H*).^13^C NMR (CDCl_3_) δ: 175.1, 174.9 (CO), 138.0, 129.8, 128.8, 126.7 (*C* and *C*H Ar), 81.5 (C *^t^*Bu), 58.8 (5-*C*), 56.5 (1′-*C*), 55.5 (4-*C*), 36.6 (3-*C*), 36.4 (5-*C*H_2_), 28.0 (CH_3_ *^t^*Bu), 19.5 (2′-*C*). MS: 319.5 [M+1]^+^. Anal. cal. for C_18_H_26_N_2_O_3_: C 67.90, H 8.23, N 8.80, found C 67.89, H 7.94, N 8.72.

### (4R*,5S*,1′S*)-5-Benzyl-4-[(1′-(*tert*-butoxycarbonyl)ethyl]amino-2-pyrrolidinone (7b)

Eluent: CH_2_Cl_2_:MeOH (40∶1). 40% (oil). HPLC *t*_R_ = 7.12 min (20 to 100% A in 20 min).^1^H NMR (CDCl_3_) δ:7.28-7.10 (m, 5H, H Ar), 5.59 (bs, 1H, 1-N*H*), 3.51(dt, 1H, *J* = 9.0, 4.6 Hz, 5-*H*), 3.15(m, 1H, 4-*H*), 3.11 (q, 1H, *J* = 7.0 Hz, 1′-*H*), 2.95 (dd, 1H, *J* = 13.5, 4.6 Hz, 5-C*H*_2_), 2.56 (dd, 1H, *J* = 13.5, 9.0 Hz, 5-C*H*_2_), 2.46 (dd, 1H, *J* = 16.8, 7.6 Hz, 3-*H*), 2.12 (dd, 1H, *J* = 16.8, 6.3 Hz, 3-*H*), 1.75 (bs, 1H, 4-N*H*), 1.40 (s, 9H, CH_3_ *^t^*Bu), 1.16 (d, 3H, *J* = 7.0, 2′-*H*).^13^C NMR (CDCl_3_) δ: 175.3, 174.9 (CO), 136.9, 129.1, 129.0, 128.6, 126.8 (*C* Ar), 81.4 (C *^t^*Bu), 62.1 (5-*C*), 57.5 (4-*C*), 54.9 (1′-*C*), 41.0 (5-*C*H_2_), 37.7 (3-*C*), 28.0 (CH_3_ *^t^*Bu), 19.4 (2′-*C*). MS: 319.4 [M+1]^+^. Anal. cal. for C_18_H_26_N_2_O_3_: C 67.90, H 8.23, N 8.80, found C 68.01, H 8.40, N 8.61.

### (4S*,5S*,1′R*)-5-Benzyl-4-[(1′-(*tert*-butoxycarbonyl)ethyl]amino-2-pyrrolidinone (7c)

Eluent: CH_2_Cl_2_:MeOH (50∶1). 48% (oil). HPLC *t*_R_ = 6.28 min (20 to 100% A in 20 min).^1^H NMR (CDCl_3_) δ:7.27–7.10 (m, 5H, H Ar), 5.35 (bs, 1H, 1-N*H*), 3.84 (dddd, 1H, *J* = 11.2, 6.5, 3.4, 0.8 Hz, 5-*H*), 3.64 (m, 1H 4-*H*), 3.28 (q, 1H, *J* = 6.9, 1′-*H*), 2.95 (dd, 1H, *J* = 13.5, 3.4 Hz, 5-C*H*_2_), 2.55 (dd, 1H, *J* = 13.5, 11.2 Hz, 5-C*H*_2_), 2.47 (dd, 1H, *J* = 16.4, 7.3 Hz, 3-*H*), 2.28 (dd, 1H, *J* = 16.4, 8.1 Hz, 3-*H*), 1.69 (bs, 1H, 4-N*H*), 1.41 (s, 9H, *^t^*Bu), 1.24 (d, 3H, *J* = 6.9, 2′-*H*).^13^C NMR (CDCl_3_) δ: 175.1, 174.8 (CO), 137.9, 129.1, 128.9, 126.8 (*C* Ar), 81.5 (C *^t^*Bu), 58.9 (5-*C*), 55.2 (1′-*C*), 54.5 (4-*C*), 36.6 (3-*C*), 36.2 (5-*C*H_2_), 28.1 (CH_3_ *^t^*Bu), 19.4 (2′-*C*). MS: 319.4 [M+1]^+^. Anal. cal. for C_18_H_26_N_2_O_3_: C 67.90, H 8.23, N 8.80, found C 67.63, H 8.10, N 8.55.

### (4S*,5S*)-5-Benzyl-4-[(*tert*-butoxycarbonyl)methyl]amino-2-pyrrolidinone (8a)

Eluent: CH_2_Cl_2_:MeOH (40∶1). 40% (oil). HPLC *t*_R_ = 6.29 min (20 to 100% A in 20 min). ^1^H NMR (CDCl_3_) δ: 7.27-7.11 (m, 5H, *H* Ar), 5.38 (bs, 1H, 1-N*H*), 3.81 (dddd, 1H, *J* = 11.1, 6.6, 3.4, 0.8 Hz, 5-*H*), 3.56 (m, 1H, *J* = 8.4, 7.5, 6.6 Hz, 4-*H*), 3.28 (bs, 2H, C*H*_2_ Gly), 2.94 (dd, 1H, *J* = 13.5, 3.5 Hz, 5-C*H*_2_), 2.56 (dd, 1H, *J* = 13.5, 11.1 Hz, 5-C*H*_2_), 2.45 (dd, 1H, *J* = 16.5, 7.5 Hz, 3-*H*), 2.25 (dd, 1H, *J* = 16.4, 8.4 Hz, 3-*H*), 1.77 (bs, 1H, 4-N*H*), 1.42 (s, 9H, CH_3_ *^t^*Bu), ^13^C NMR (CDCl_3_) δ: 174.8, 171.1 (CO), 137.7, 129.1, 128.9, 126.8 (*C* and *C*H Ar), 81.3 (C *^t^*Bu), 58.7 (5-*C*), 56.3 (4-*C*), 50.0 (*C*H_2_ Gly), 36.4 (3-*C*), 36.2 (5-*C*H_2_), 28.1 (CH_3_ *^t^*Bu). MS: 305.5 [M+1]^+^. Anal. cal. for C_17_H_24_N_2_O_3_: C 67.08, H 7.95, N 9.20, found C 66.86, H 8.08, N 8.97.

### (4R*,5S*)-5-Benzyl-4-[(*tert*-butoxycarbonyl)methyl]amino-2-pyrrolidinone (8b)

Eluent: CH_2_Cl_2_:MeOH (40∶1). 43% (oil).HPLC *t*_R_ = 7.23 min (20 to 100% A in 20 min). ^1^H NMR (CDCl_3_) δ: 7.35–7.18 (m, 5H, H Ar), 5.80 (bs, 1H, 1-N*H*), 3.67 (m, 1H, 5-*H*), 3.26 (m, 3H, C*H*_2_ Gly, 4-*H*), 3.00 (dd, 1H, *J* = 13.6, 4.9 Hz, 5-C*H*_2_), 2.67 (dd, 1H, *J* = 13.6, 8.8 Hz, 5-C*H*_2_), 2.59 (dd, 1H, *J* = 17.0, 7.6 Hz, 3-*H*), 2.21 (dd, 1H, *J* = 17.0, 5.4 Hz, 3-*H*), 1.46 (s, 9H, CH_3_ *^t^*Bu). ^13^C NMR (CDCl_3_) δ: 175.1, 171.1 (CO), 137.1, 129.0, 128.9, 127.0 (*C* and *C*H Ar), 81.8 (C *^t^*Bu), 61.89 (5-*C*), 58.8 (4-*C*), 49.3 (*C*H_2_ Gly), 41.3 (5-*C*H_2_), 37.5 (3-*C*), 28.0 (CH_3_ *^t^*Bu). MS: 305.4 [M+1]^+^. Anal. cal. for C_17_H_24_N_2_O_3_: C 67.08, H 7.95, N 9.20, found C 66.98, H 7.69, N 8.79.

## Supporting Information

Figure S1 **Chiral HPLC chromatograms for 1 and 3a.**
(PDF)

Figure S2 **Chiral HPLC chromatograms for 4a and 6a.**
(PDF)

Figure S3 **^1^H NMR spectra for monitoring the intermediate formation.**
(PDF)

Figure S4 **^1^H NMR spectra of deuterated diamino esters 3a–3c.**
(PDF)

Figures S5 **^1^H NMR spectra of deuterated diamino esters 4a–4b.**
(PDF)

Figure S6 **X-Ray packing of compound 7a.**
(PDF)

Figure S7 **X-Ray packing of compound 7b.**
(PDF)

Figure S8 **NMR Spectra for new compounds 2–8**
(PDF)

Table S1 **Direct reductive amination trials.**
(PDF)

Table S2 **Indirect reductive amination experiments.**
(PDF)
