# Supplementary material for: Highly Functionalized 1,2–Diamino Compounds through Reductive Amination of Amino Acid-Derived β–Keto Esters
Source: PLoS One. 2013 Jan 7;8(1):e53231. doi: 10.1371/journal.pone.0053231 (PMC3538761; doi:10.1371/journal.pone.0053231)

Figure S1. Chiral HPLC chromatograms for the starting β–ketoester and diastereoisomer **3a**

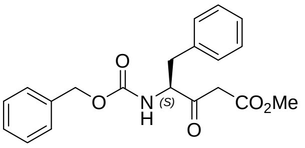

(1)

95% hexane- 5% ethanol

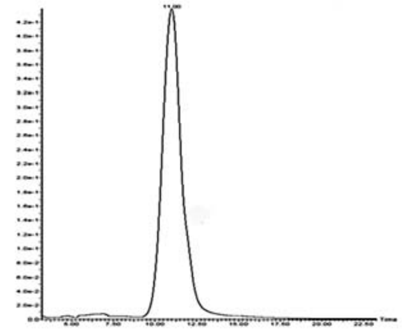

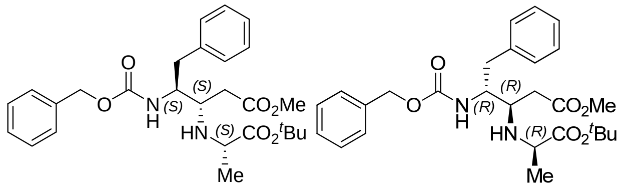

Diastereomer 3a (3*S,4*S**,1'*S**).**

99% hexane- 1% ethanol

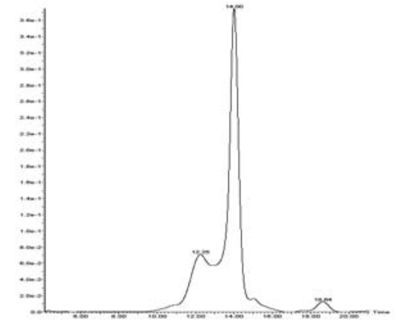

Supplement: Figure S1 — Chiral HPLC chromatograms for 1 and 3a. (PDF) [file pone.0053231.s001.pdf]
