# Supplementary material for: Highly Functionalized 1,2–Diamino Compounds through Reductive Amination of Amino Acid-Derived β–Keto Esters
Source: PLoS One. 2013 Jan 7;8(1):e53231. doi: 10.1371/journal.pone.0053231 (PMC3538761; doi:10.1371/journal.pone.0053231)

Figure S2. Chiral HPLC chromatograms for compounds **4a** and **6a**

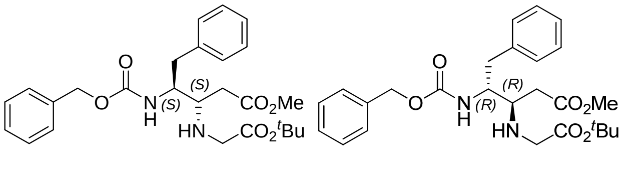

Diastereomer 4a (3*S,4*S**).**

60% hexane- 40% MTBE (methyl-tert-butylether)

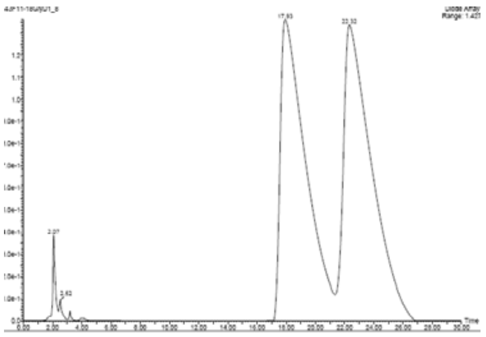

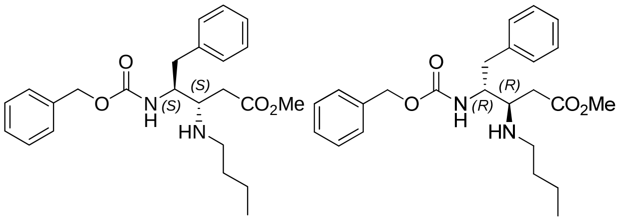

Diastereomer 6a (3*S,4*S**).**

50% hexane- 50% MTBE

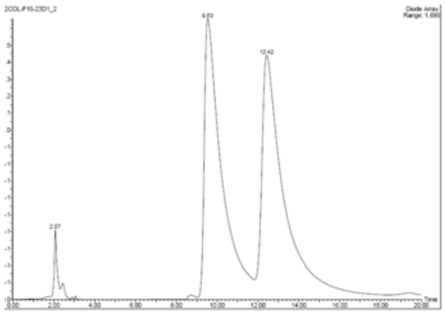

Supplement: Figure S2 — Chiral HPLC chromatograms for 4a and 6a. (PDF) [file pone.0053231.s002.pdf]
