# Supplementary material for: Highly Functionalized 1,2–Diamino Compounds through Reductive Amination of Amino Acid-Derived β–Keto Esters
Source: PLoS One. 2013 Jan 7;8(1):e53231. doi: 10.1371/journal.pone.0053231 (PMC3538761; doi:10.1371/journal.pone.0053231)

**Figure S3.** Sections of the 1H NMR spectra containing the anmine protons. A: Reaction of **1** and H-Ala-OtBu. B: Reaction of **1** and H-Gly-OtBu

A. Detail of the enamine protons in the reaction between **1** and H-Ala-O$^t$Bu ($CHCl_3$/AcOH)

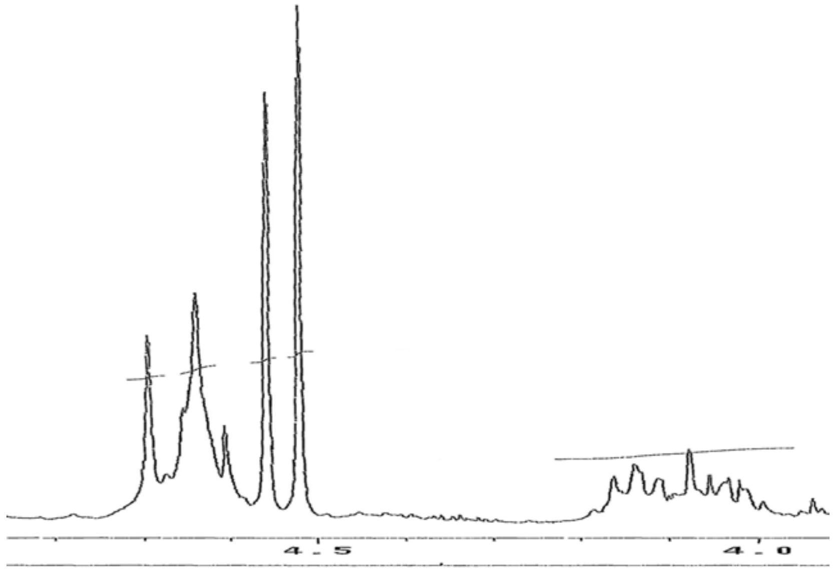


B. Detail of the enamine protons in the reaction between **1** and H-Gly-O$^t$Bu ($CHCl_3$/AcOH)

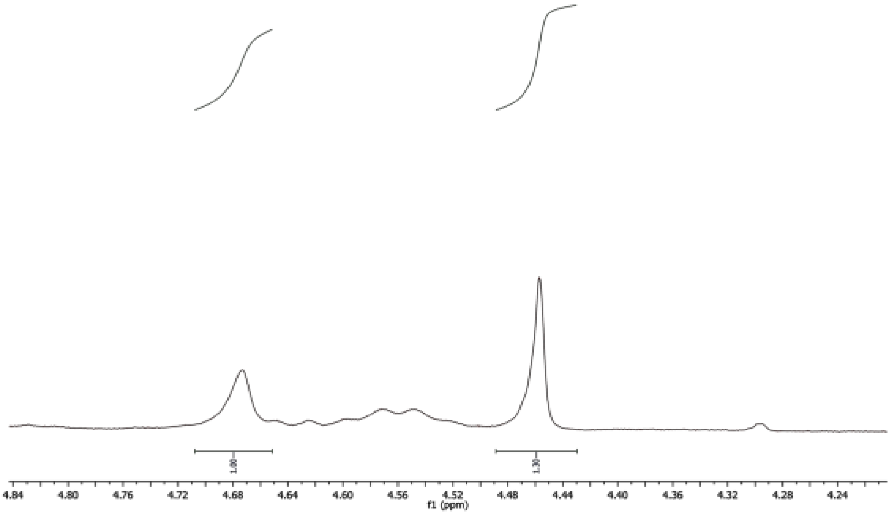

Supplement: Figure S3 — 1H NMR spectra for monitoring the intermediate formation. (PDF) [file pone.0053231.s003.pdf]
