# Supplementary figures and images for: Highly Functionalized 1,2–Diamino Compounds through Reductive Amination of Amino Acid-Derived β–Keto Esters
Source: PLoS One. 2013 Jan 7;8(1):e53231. doi: 10.1371/journal.pone.0053231 (PMC3538761; doi:10.1371/journal.pone.0053231)

**Figure S4.**  $^1\text{H}$  NMR spectra of deuterated **3a**, **3b** and **3c**.

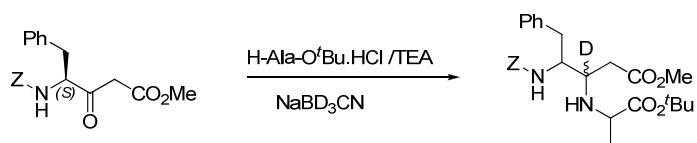

**D3a**

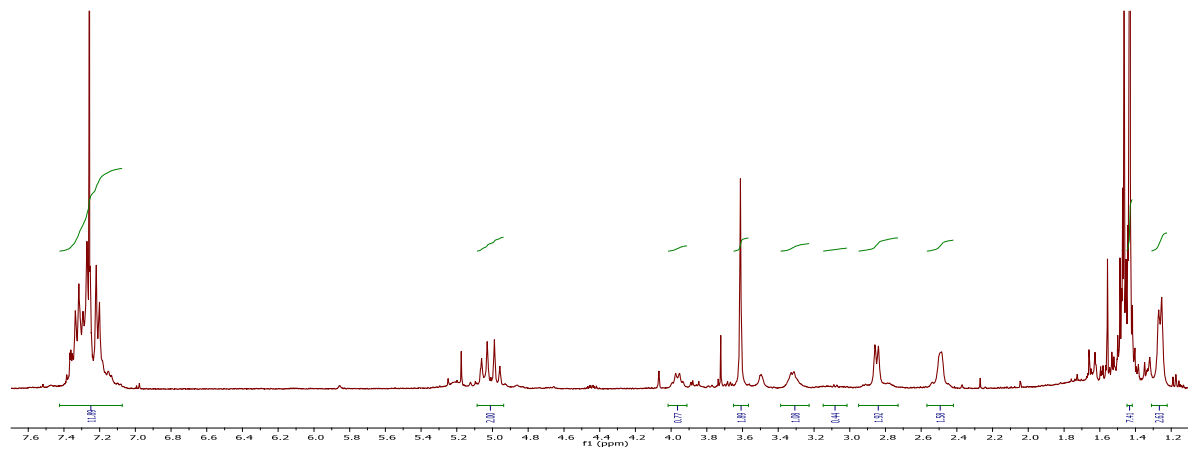

**D3b**

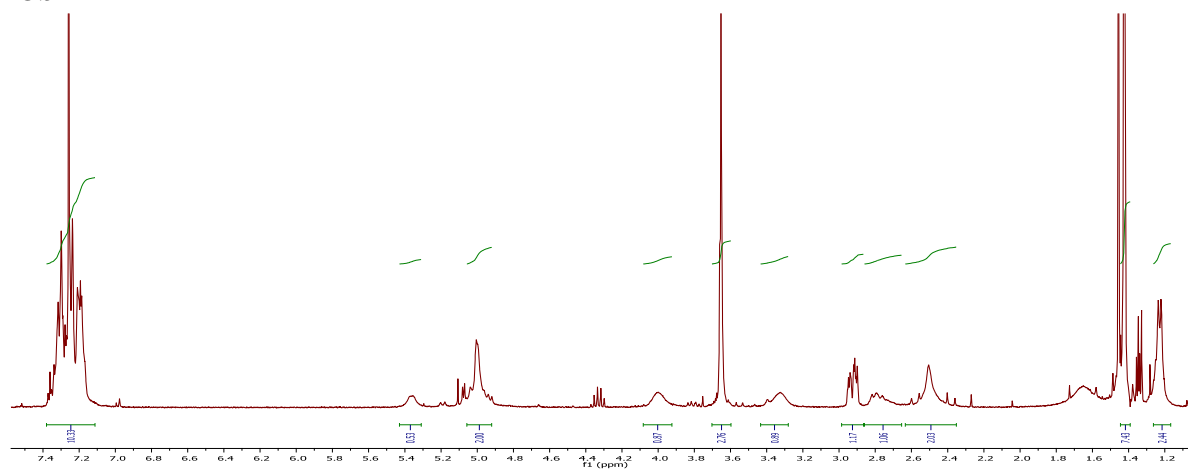

**D3c**

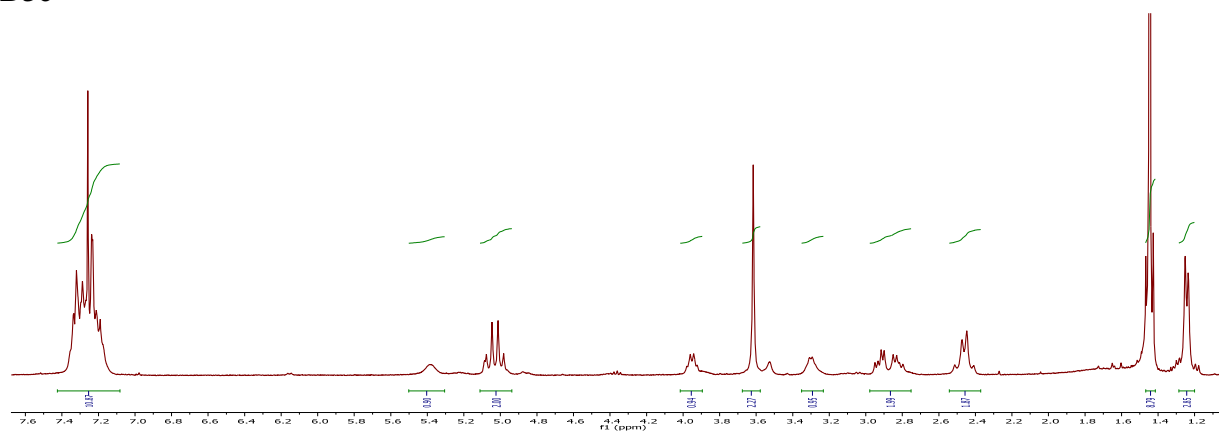

Supplement: Figure S4 — 1H NMR spectra of deuterated diamino esters 3a–3c. (PDF) [file pone.0053231.s004.pdf]

**Figure S5.**  $^1\text{H}$  NMR spectrum for deuterated **4a** and **4b**

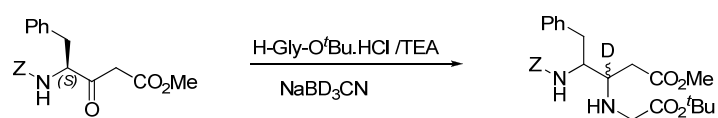

**D4a**

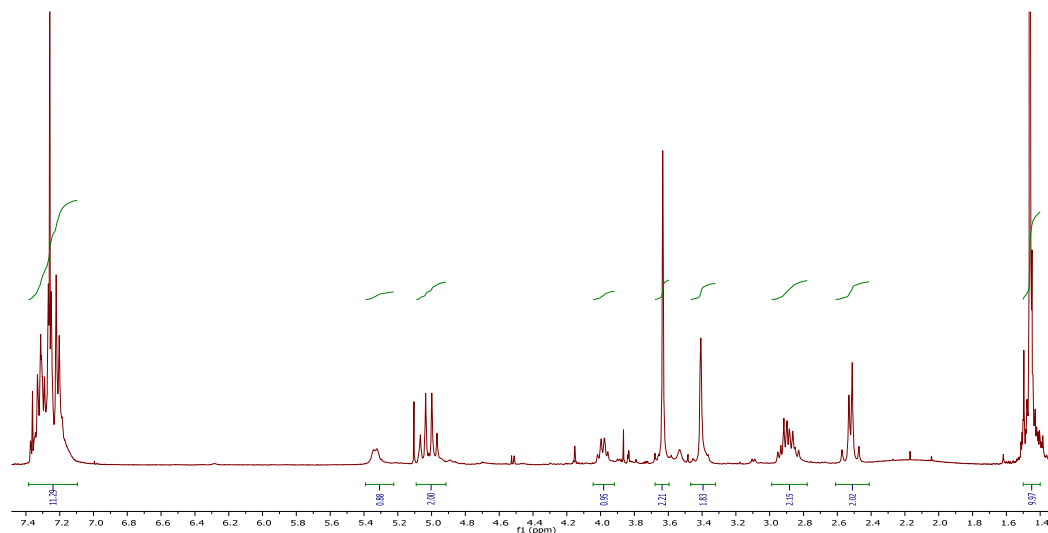

**D4b**

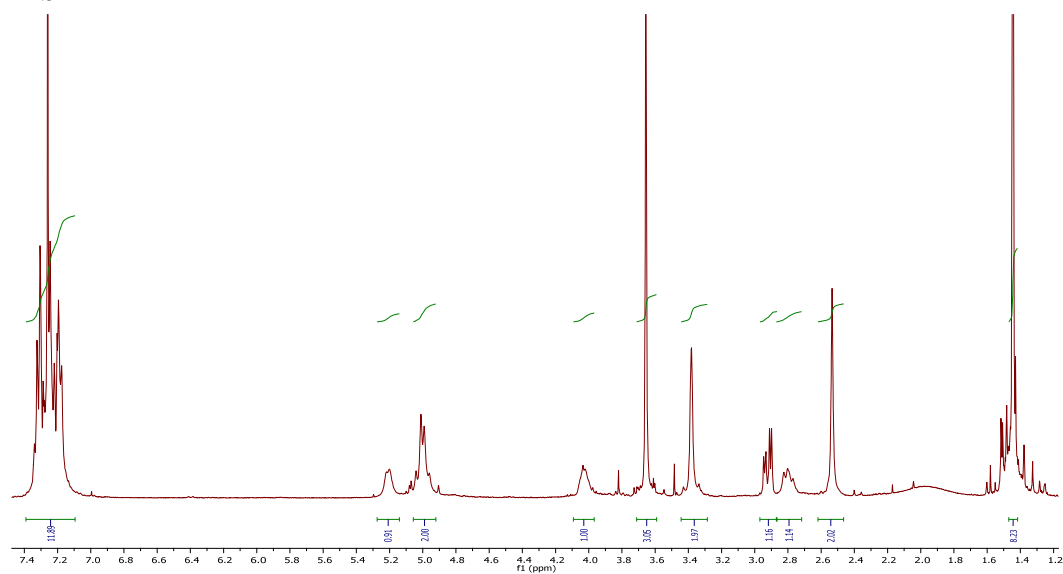

Supplement: Figures S5 — 1H NMR spectra of deuterated diamino esters 4a–4b. (PDF) [file pone.0053231.s005.pdf]

**Figure S8. NMR Spectra of new compounds**

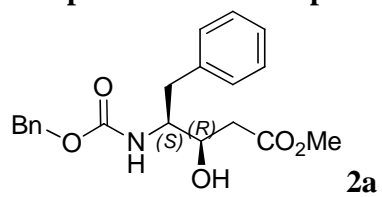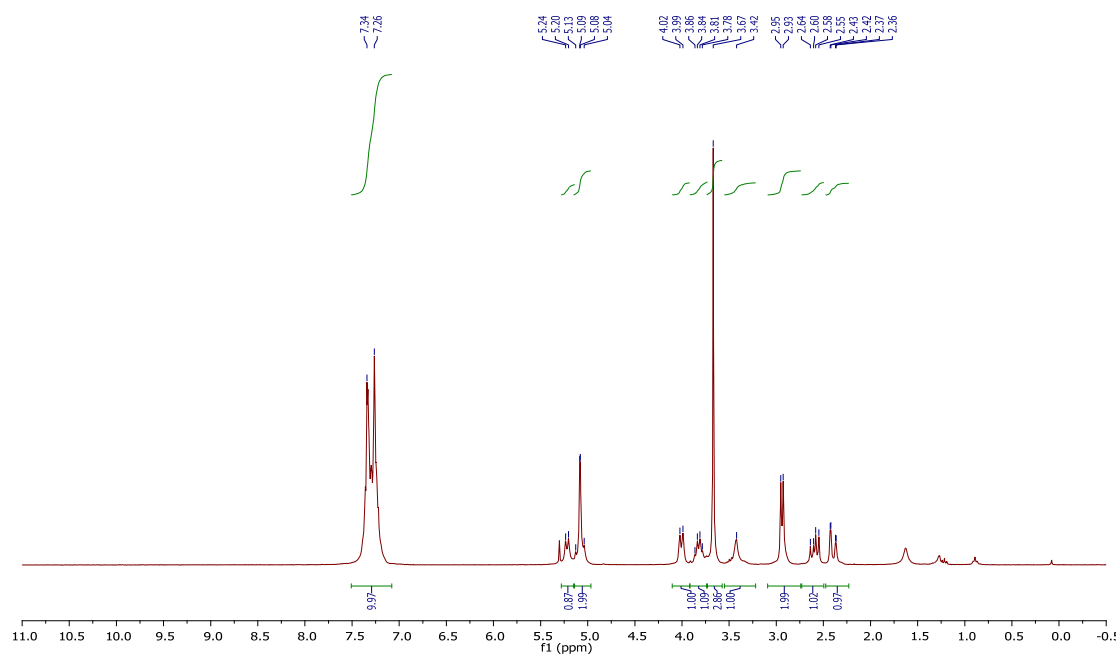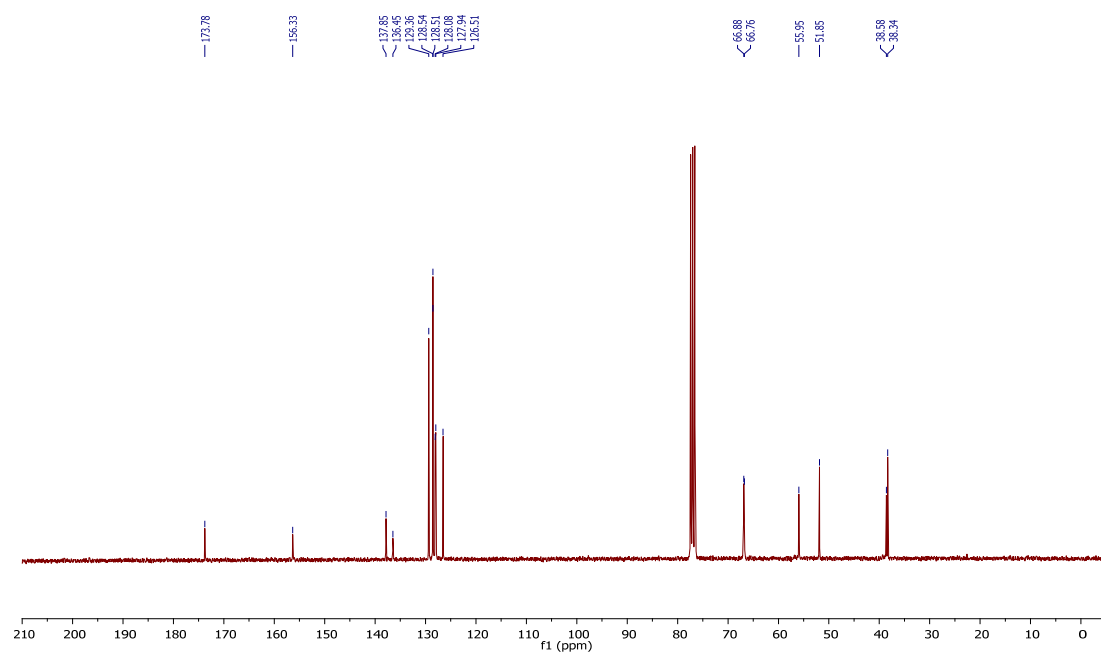

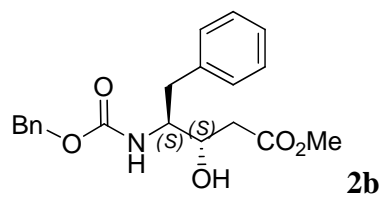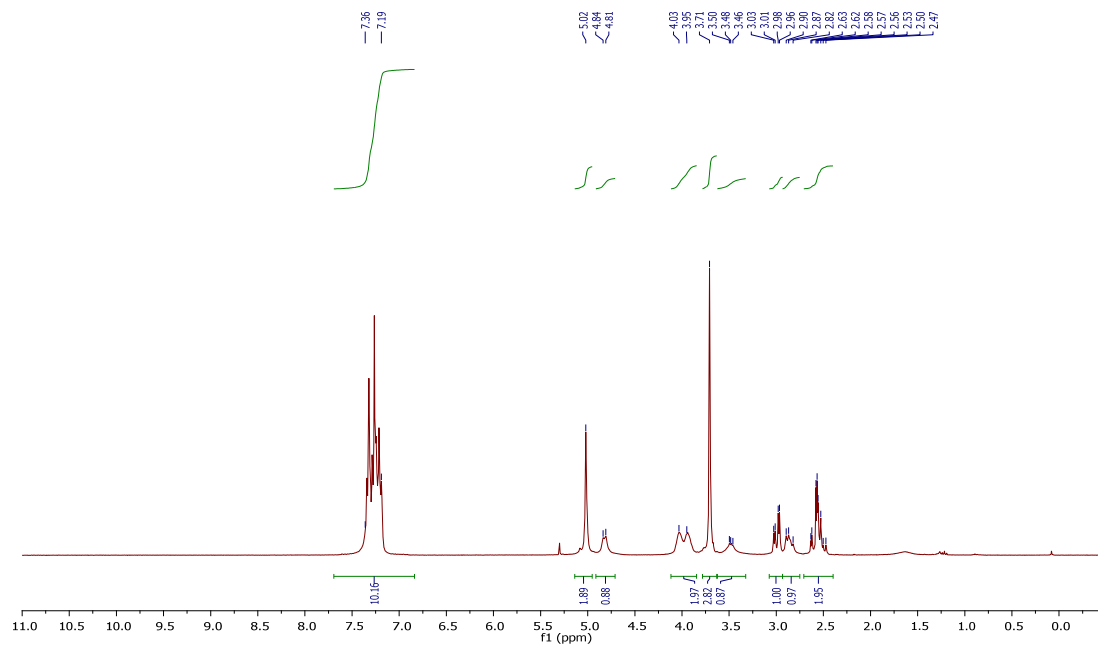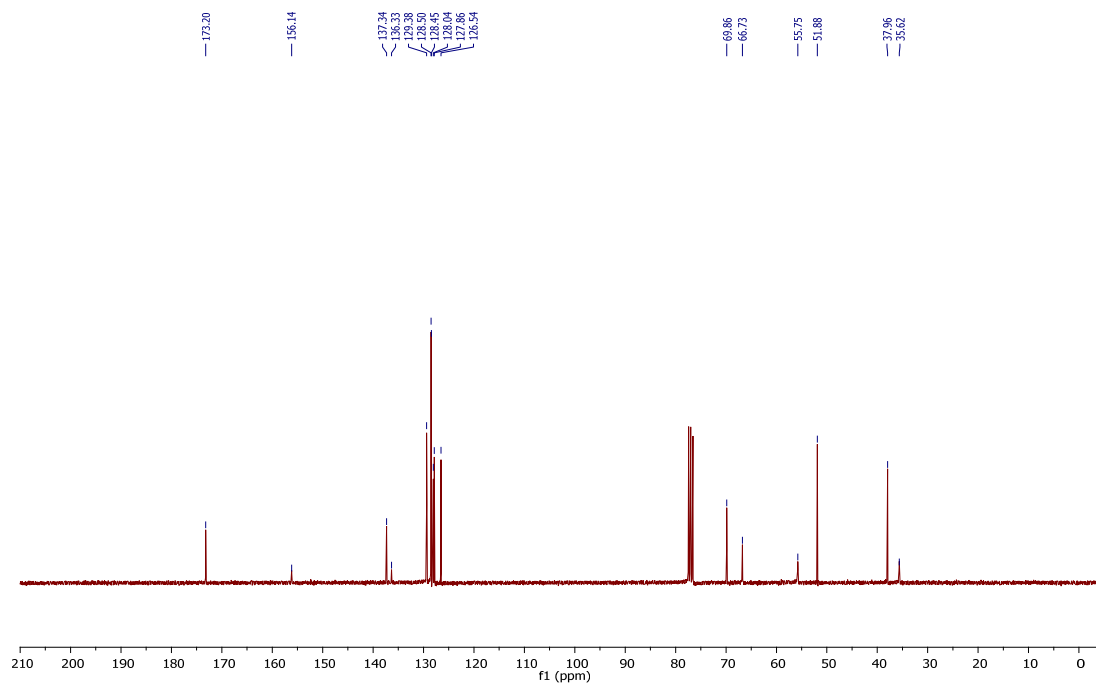

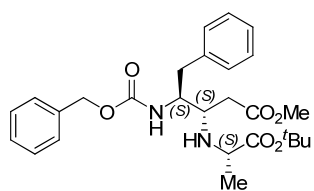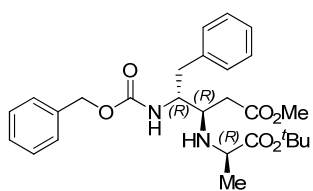

**3a**

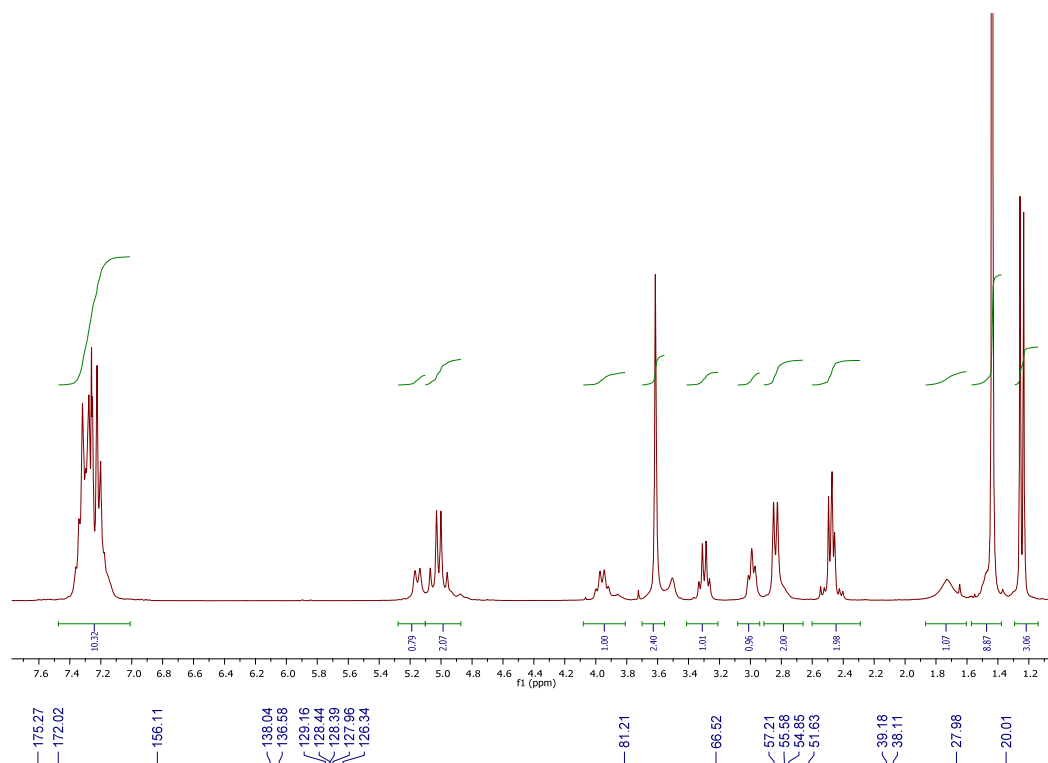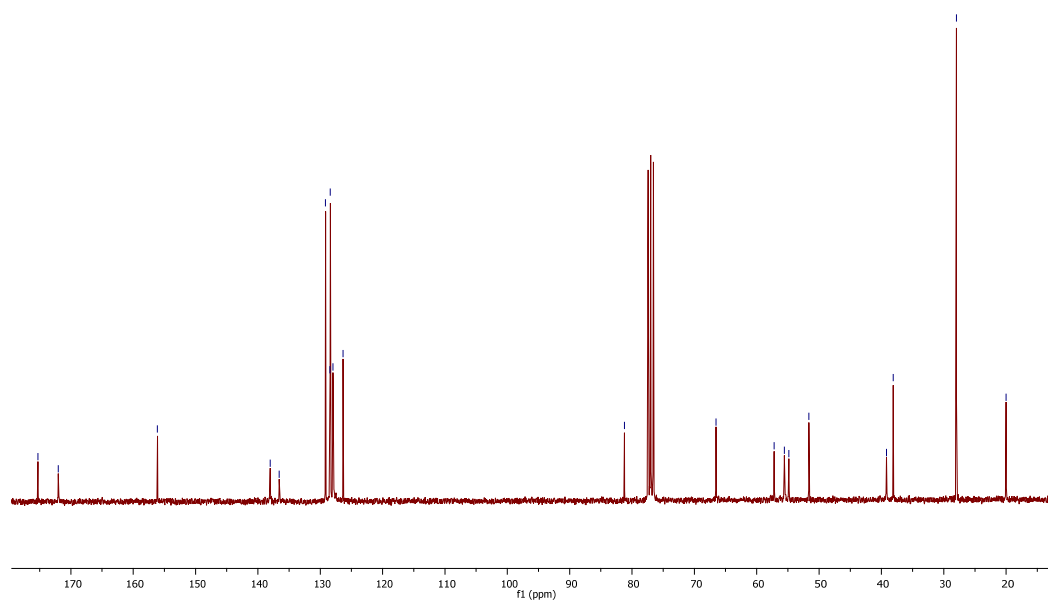

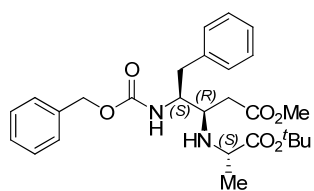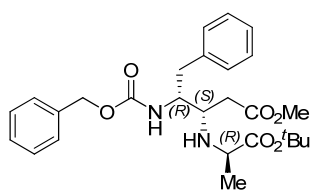

**3b**

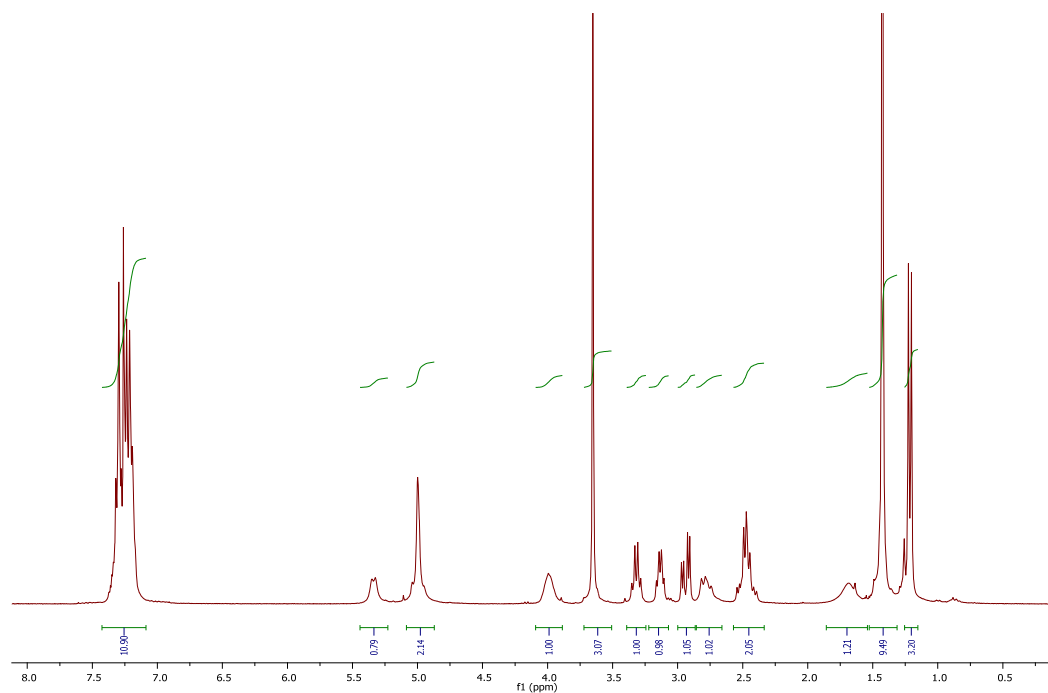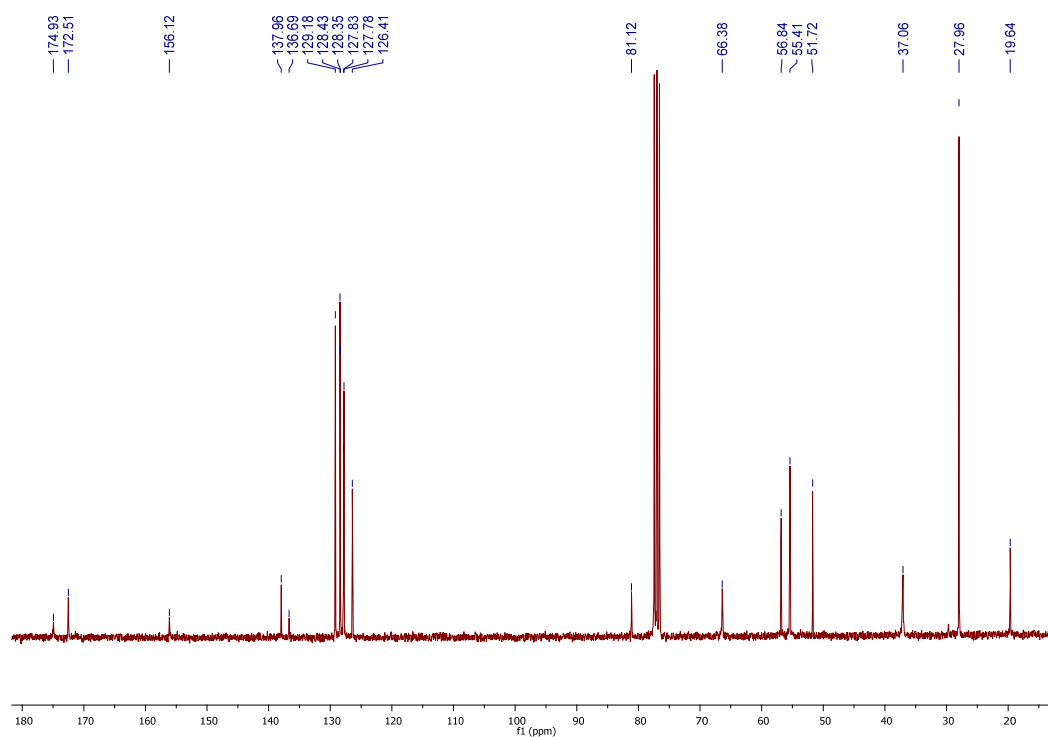

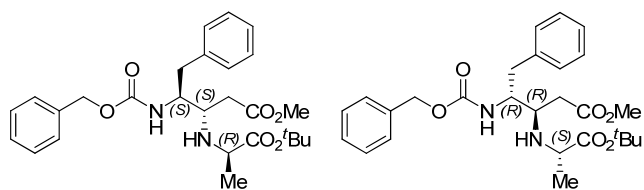

**3c**

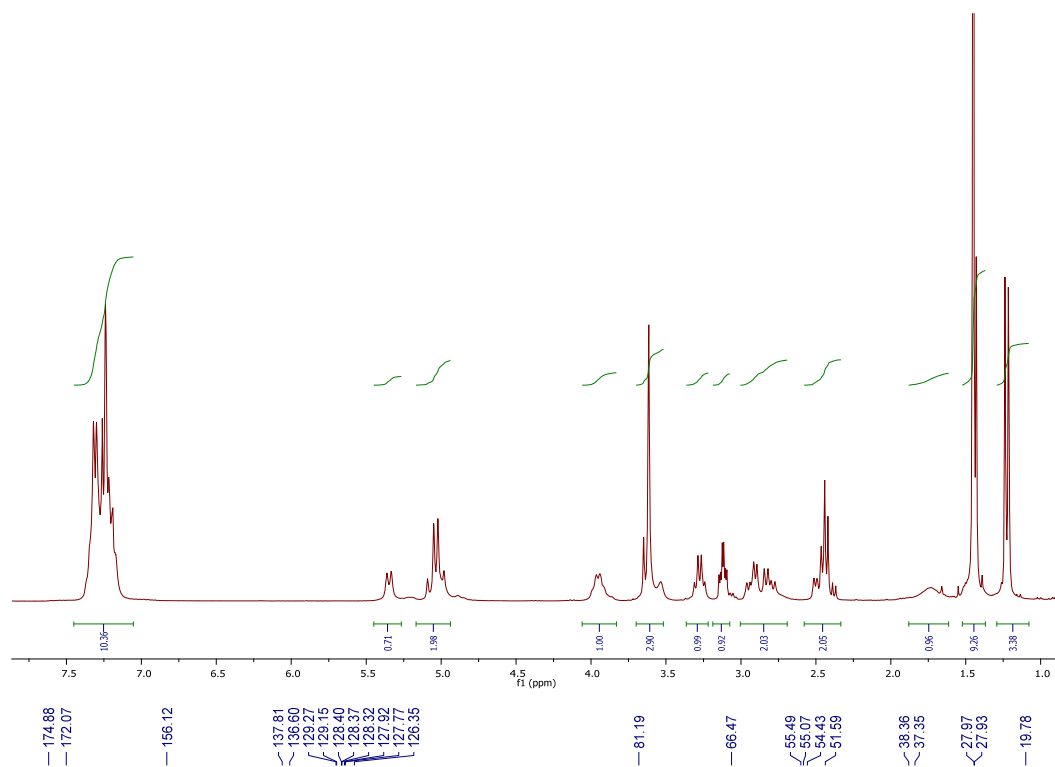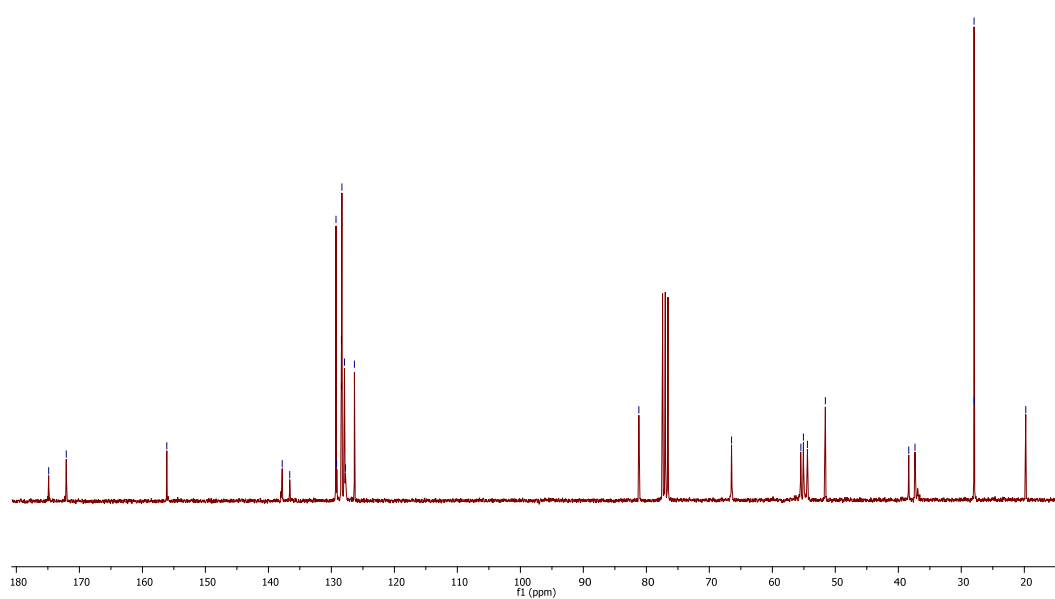

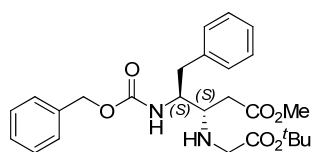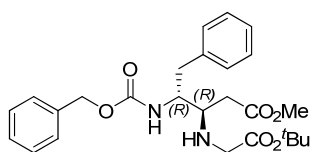

**4a**

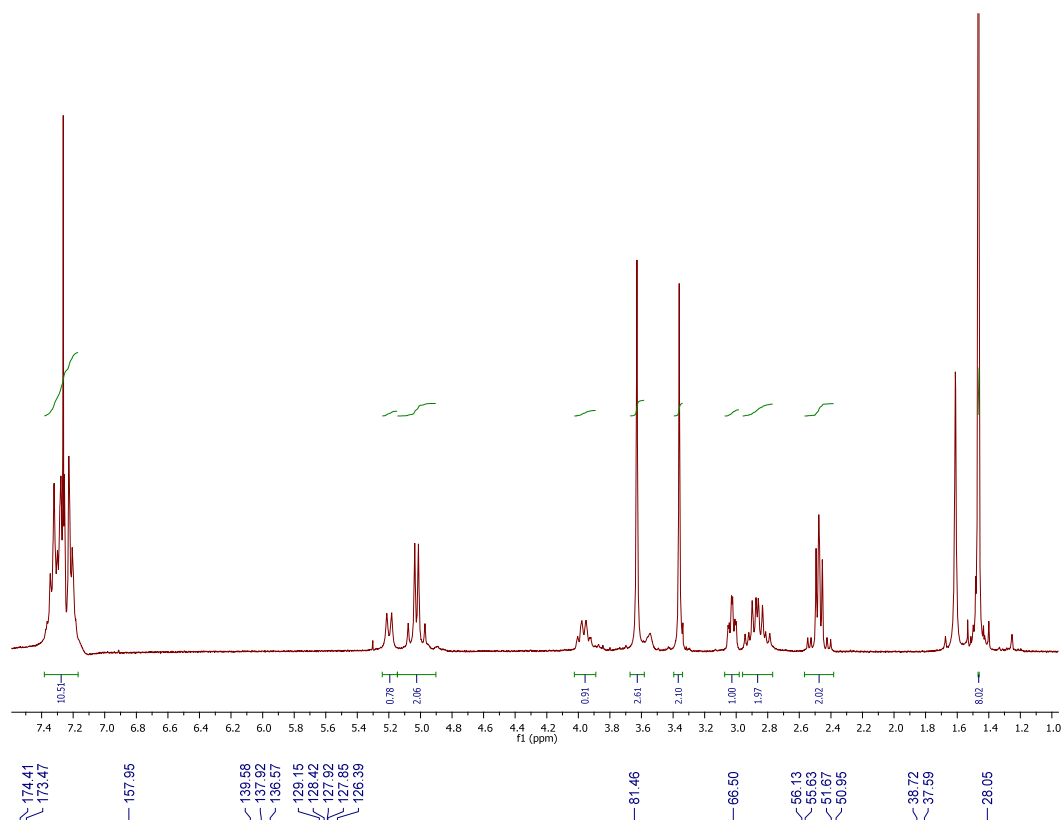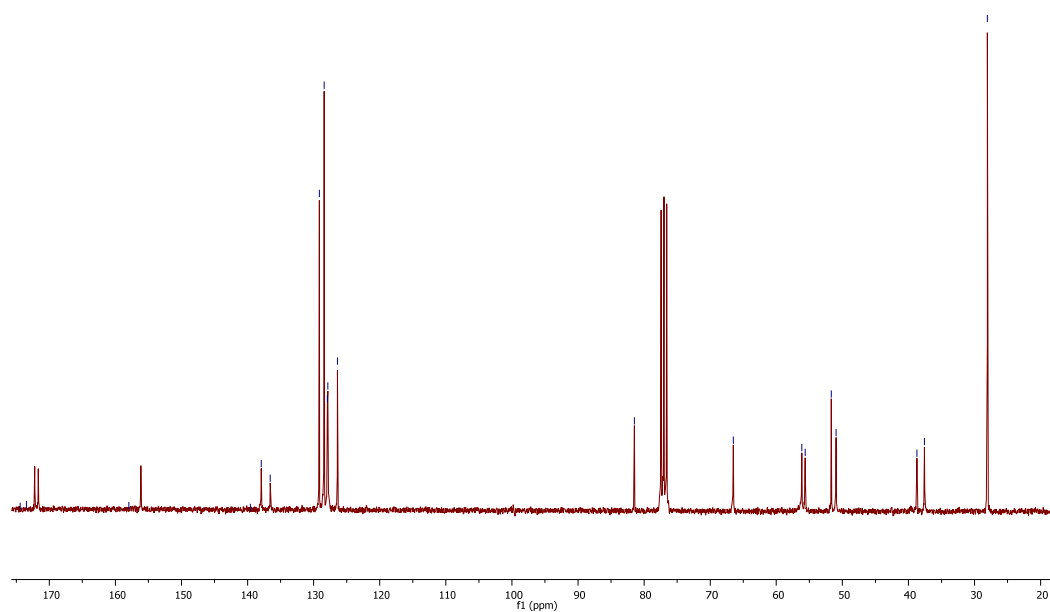

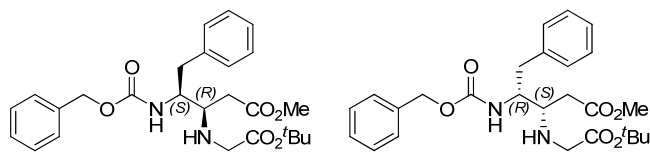

**4b**

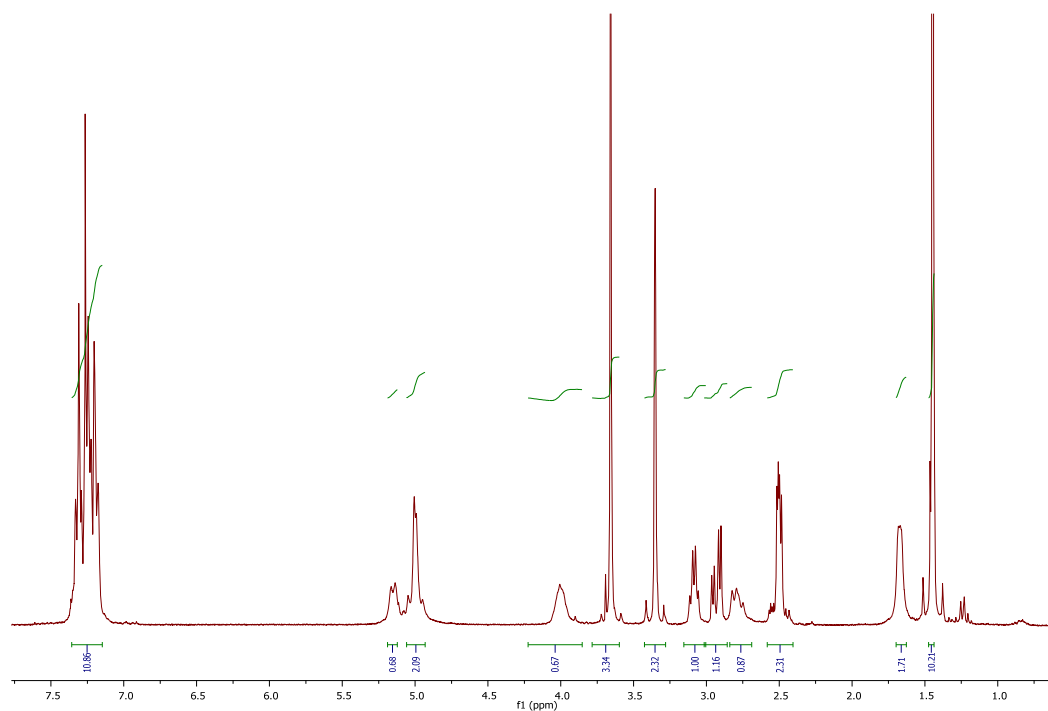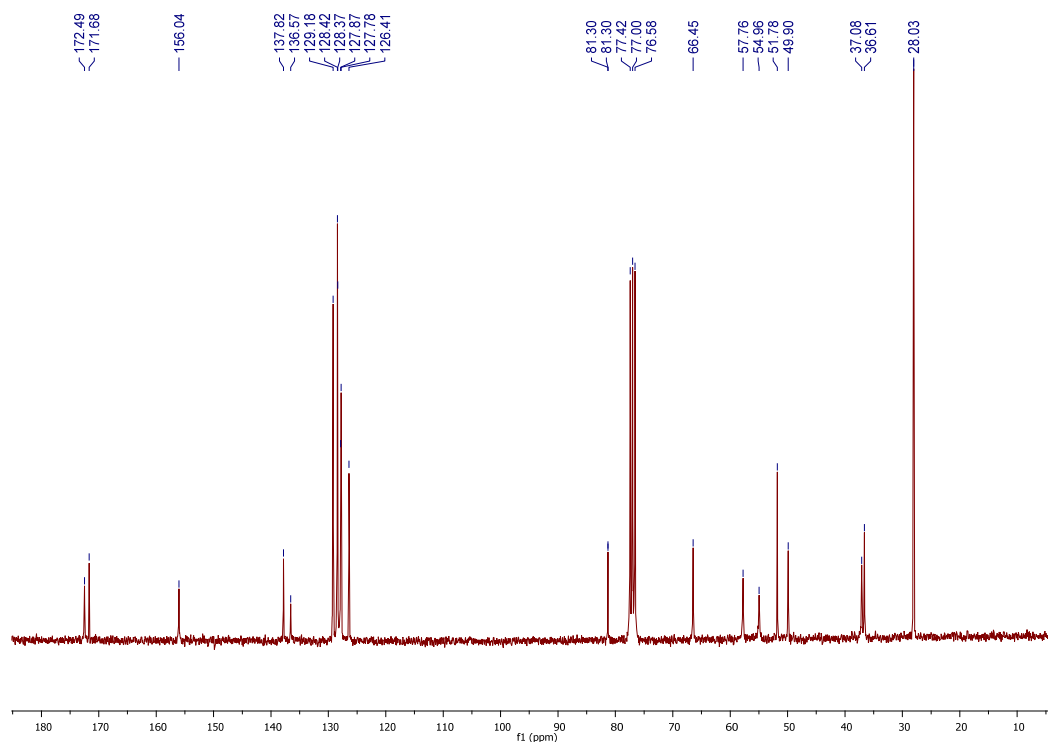

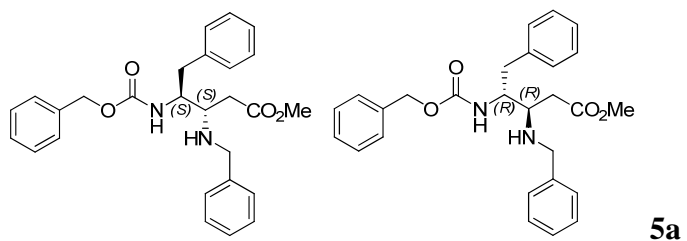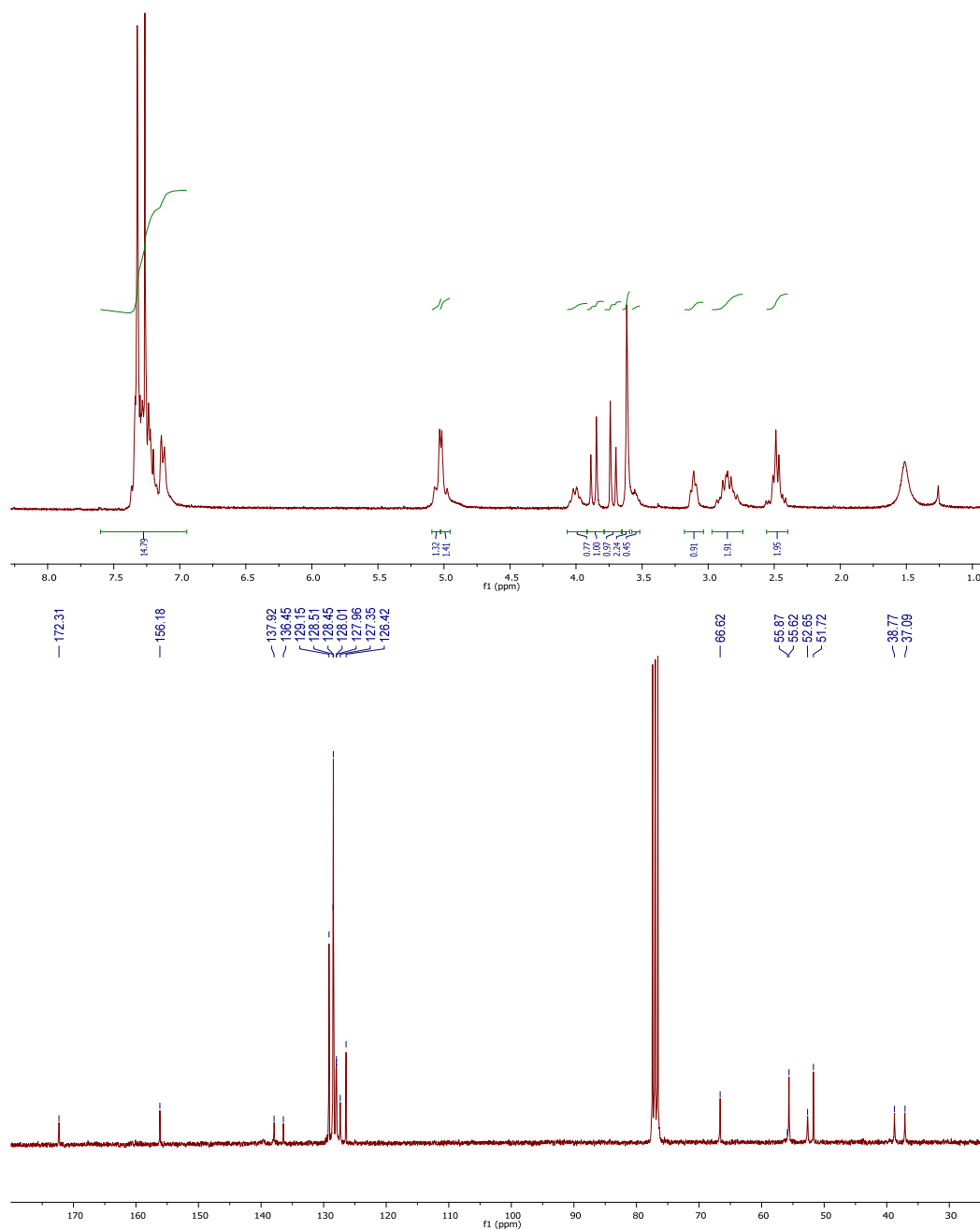

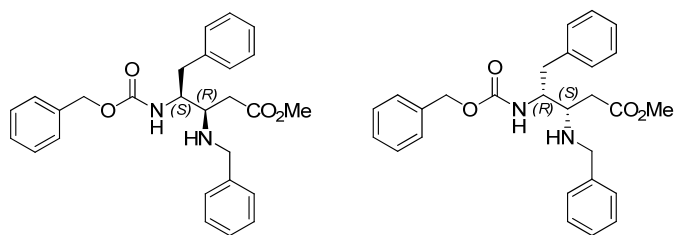

**5b**

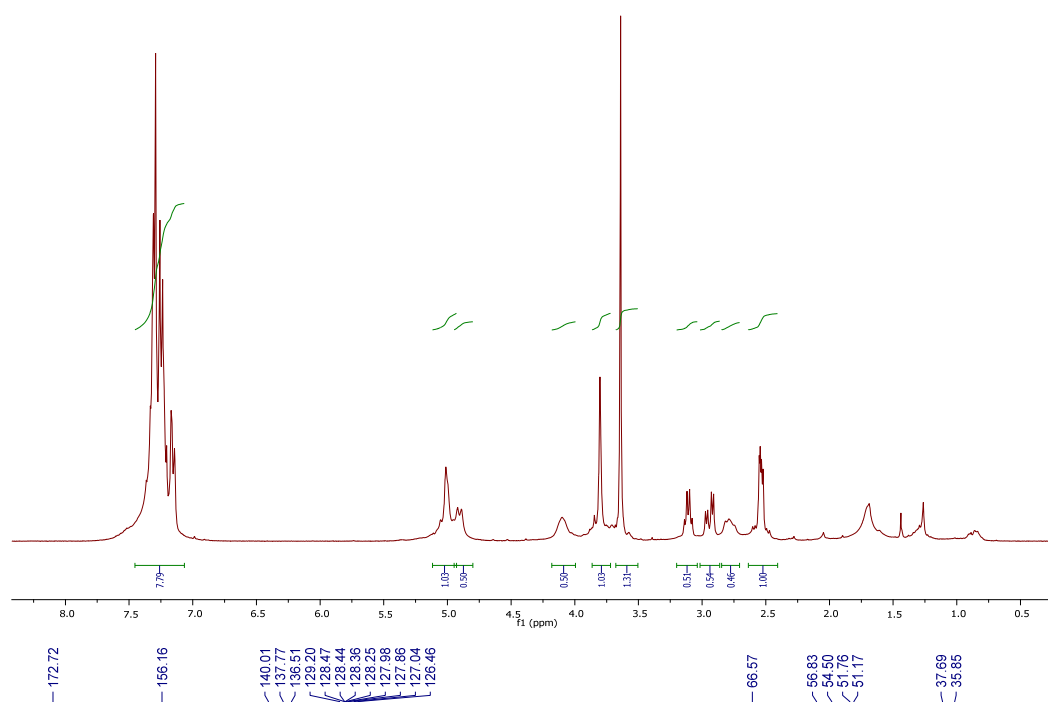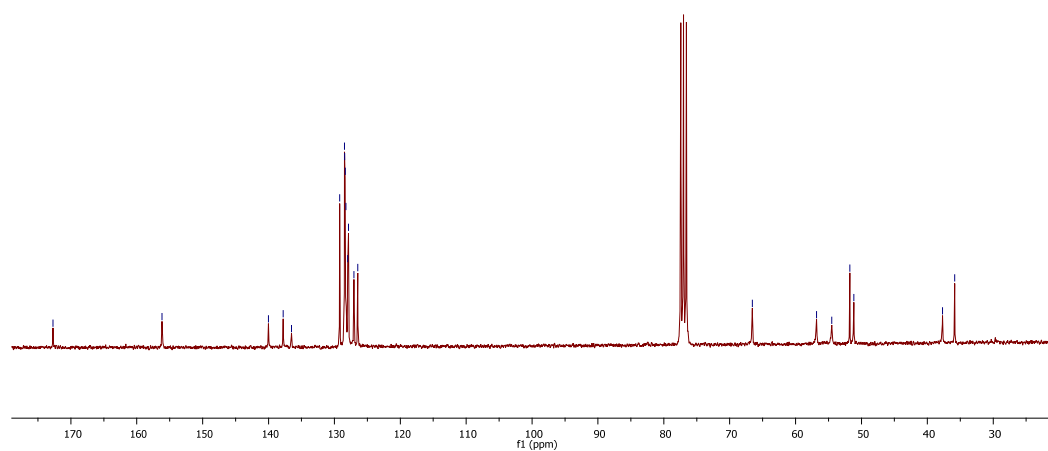

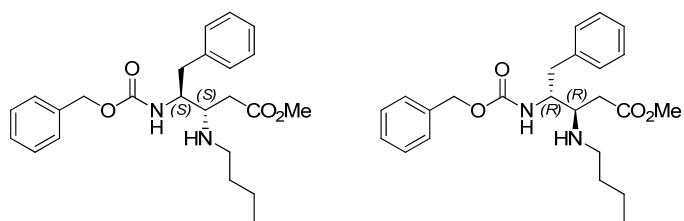

**6a**

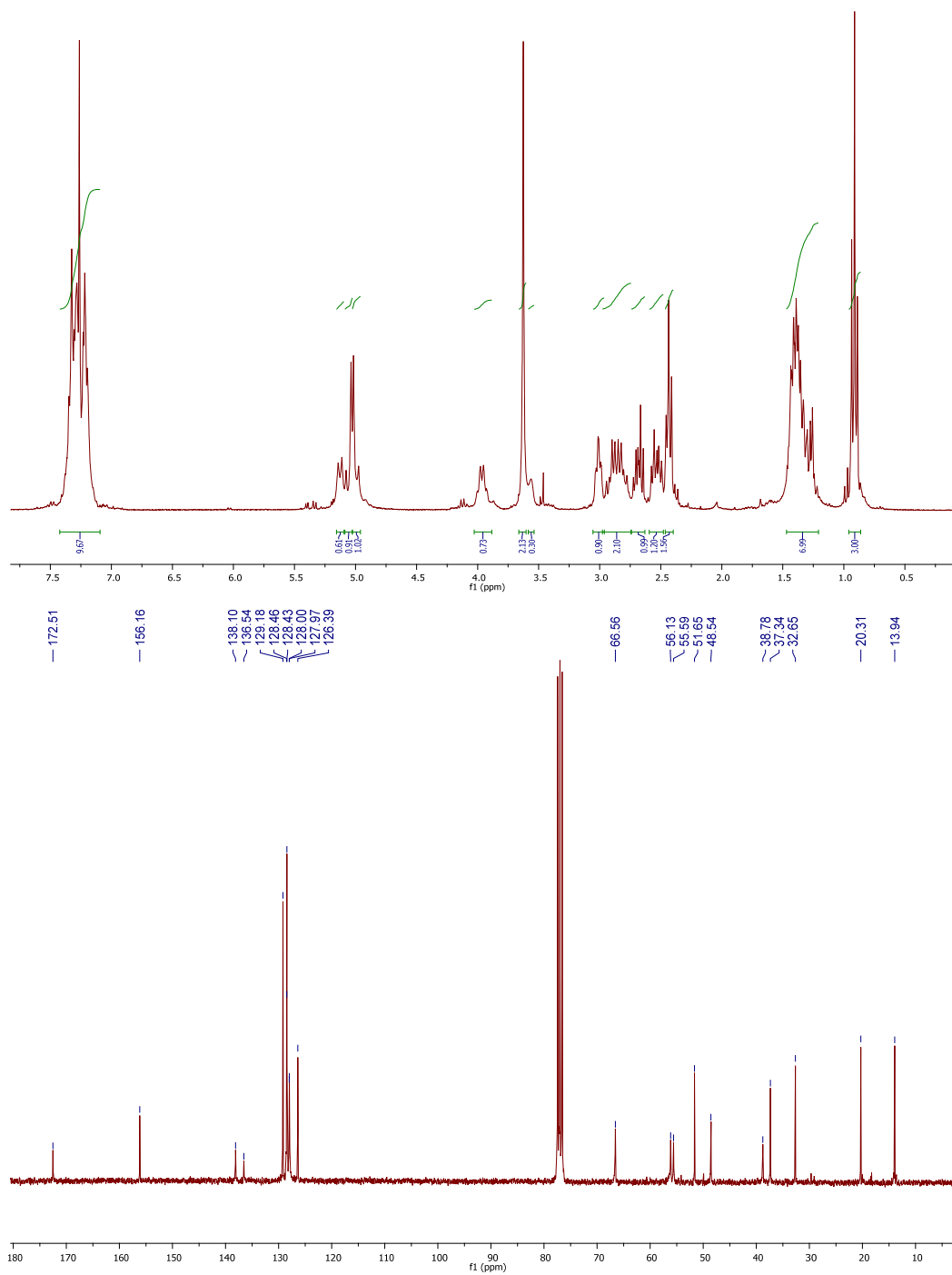

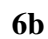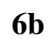

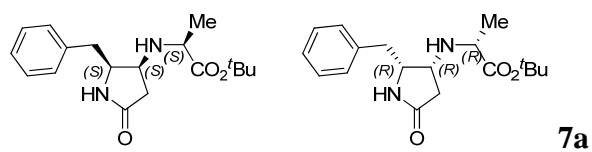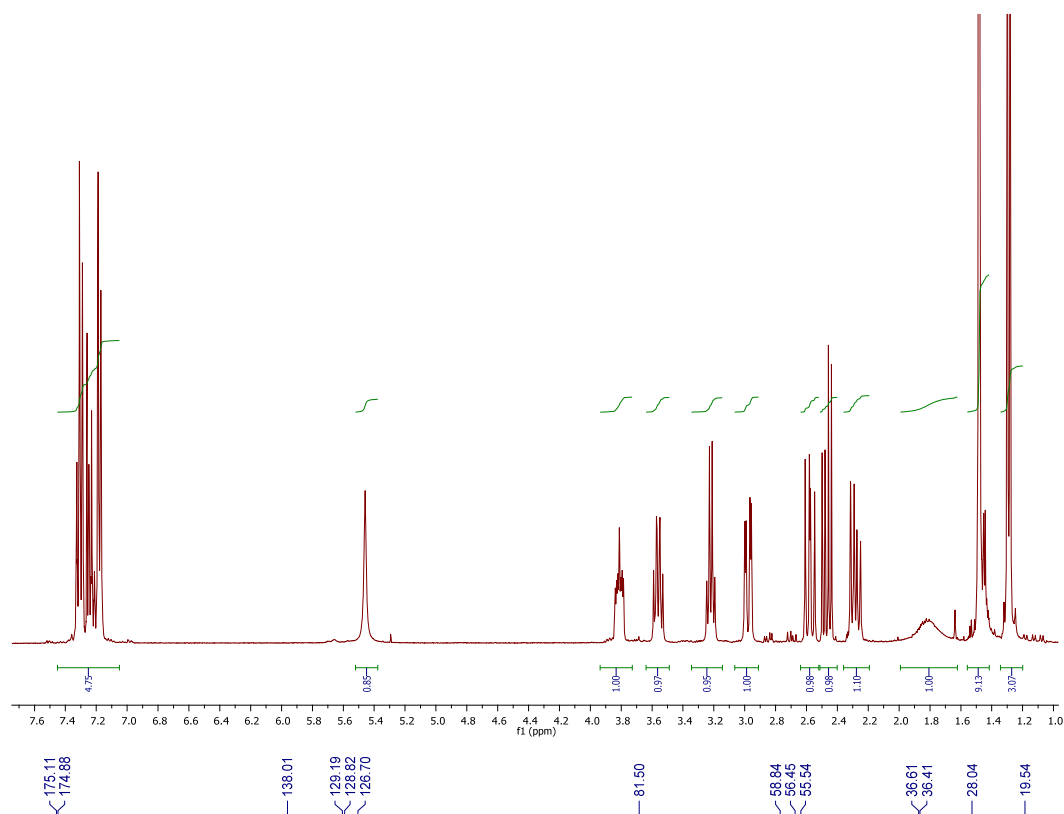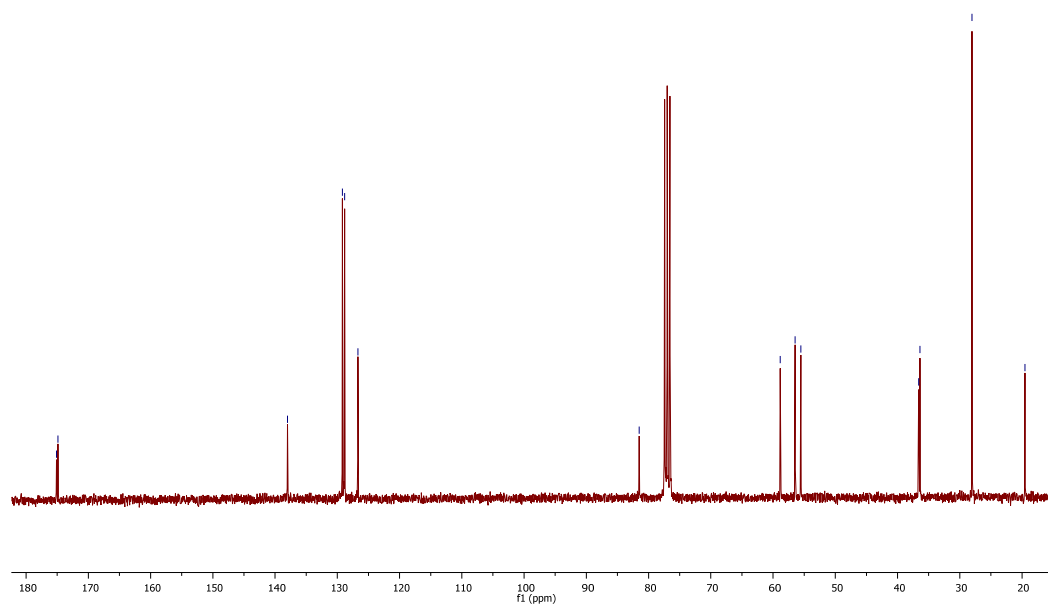

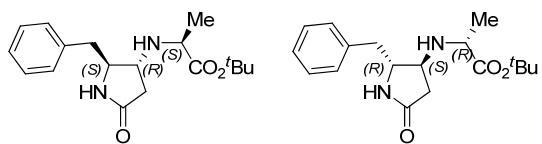

7b

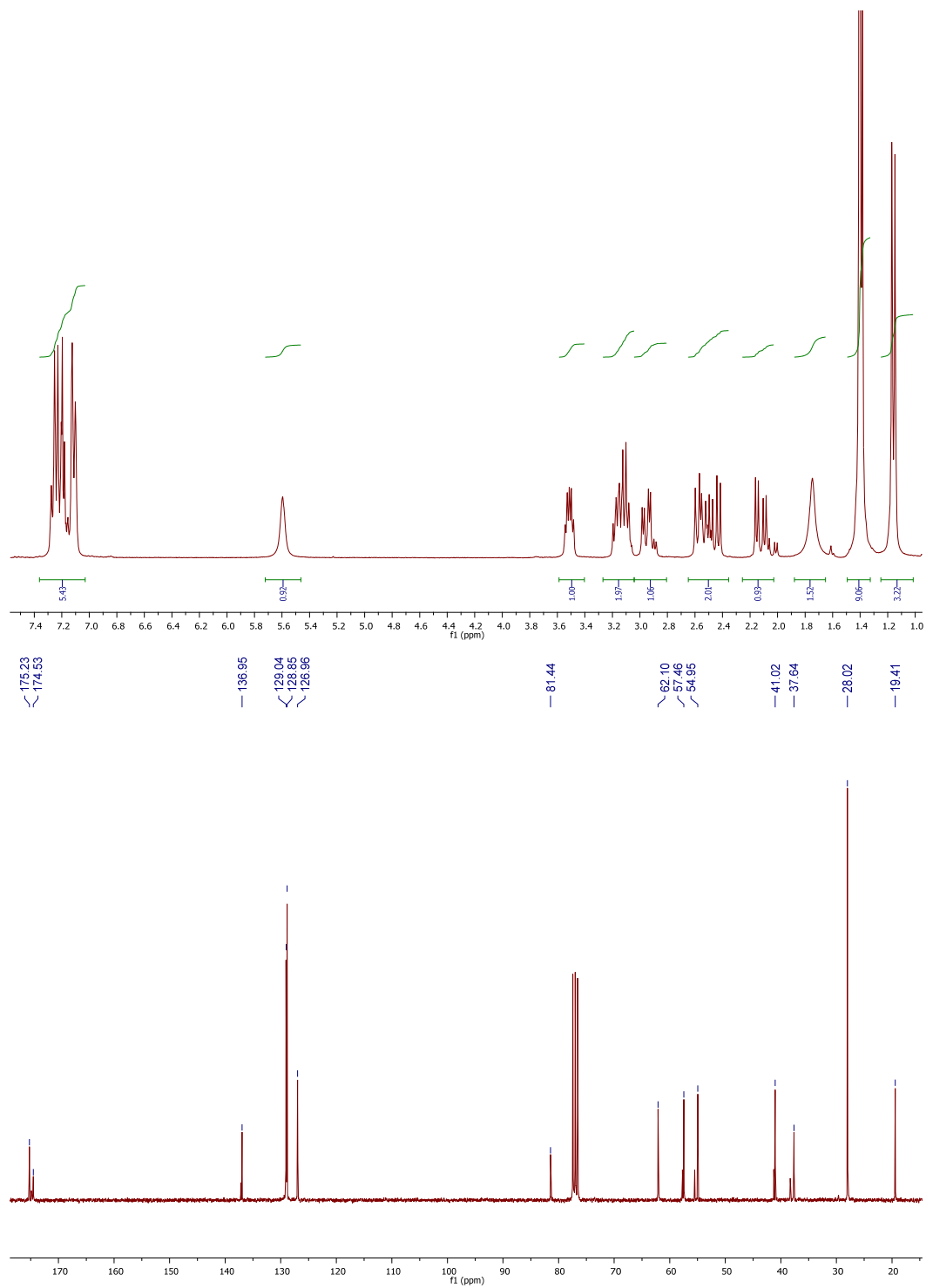

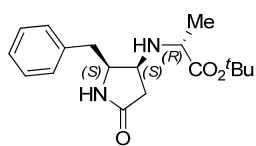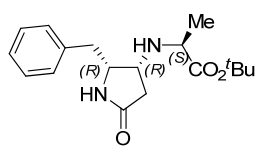

**7c**

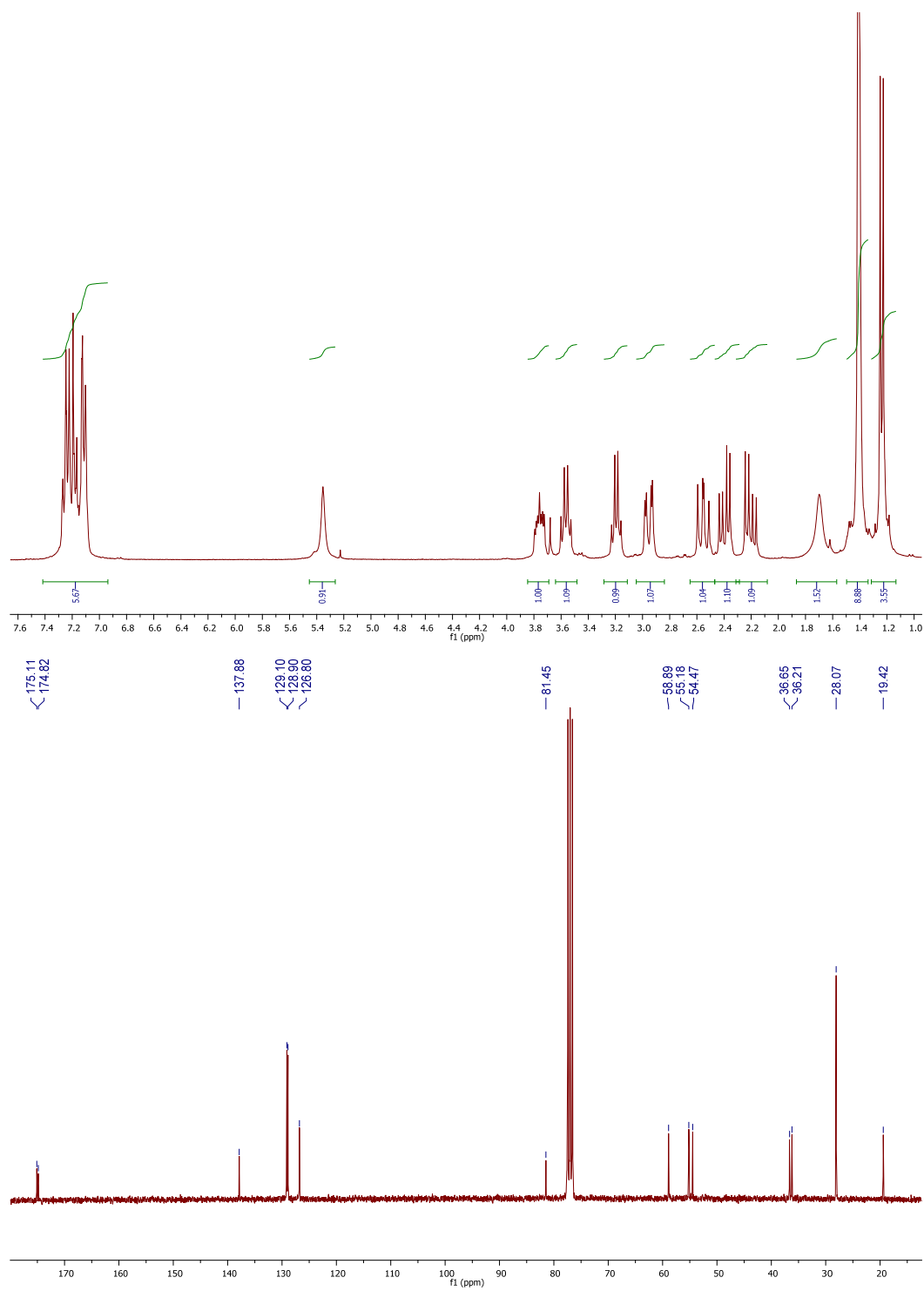

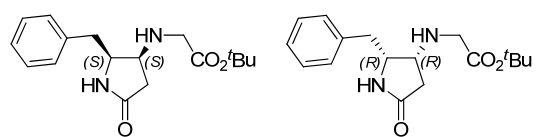

8a

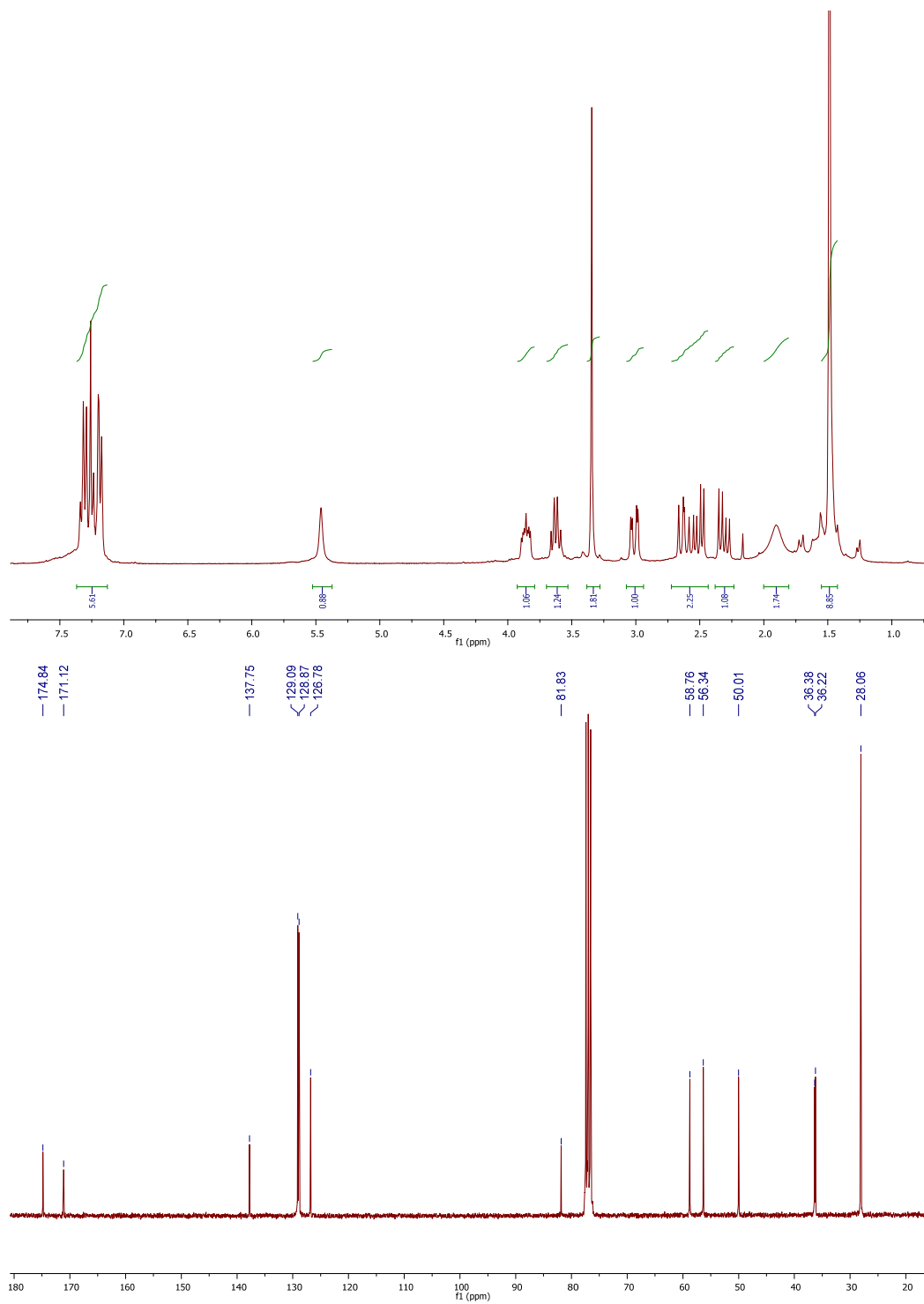

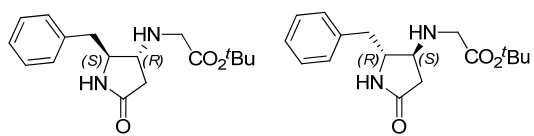

**8b**

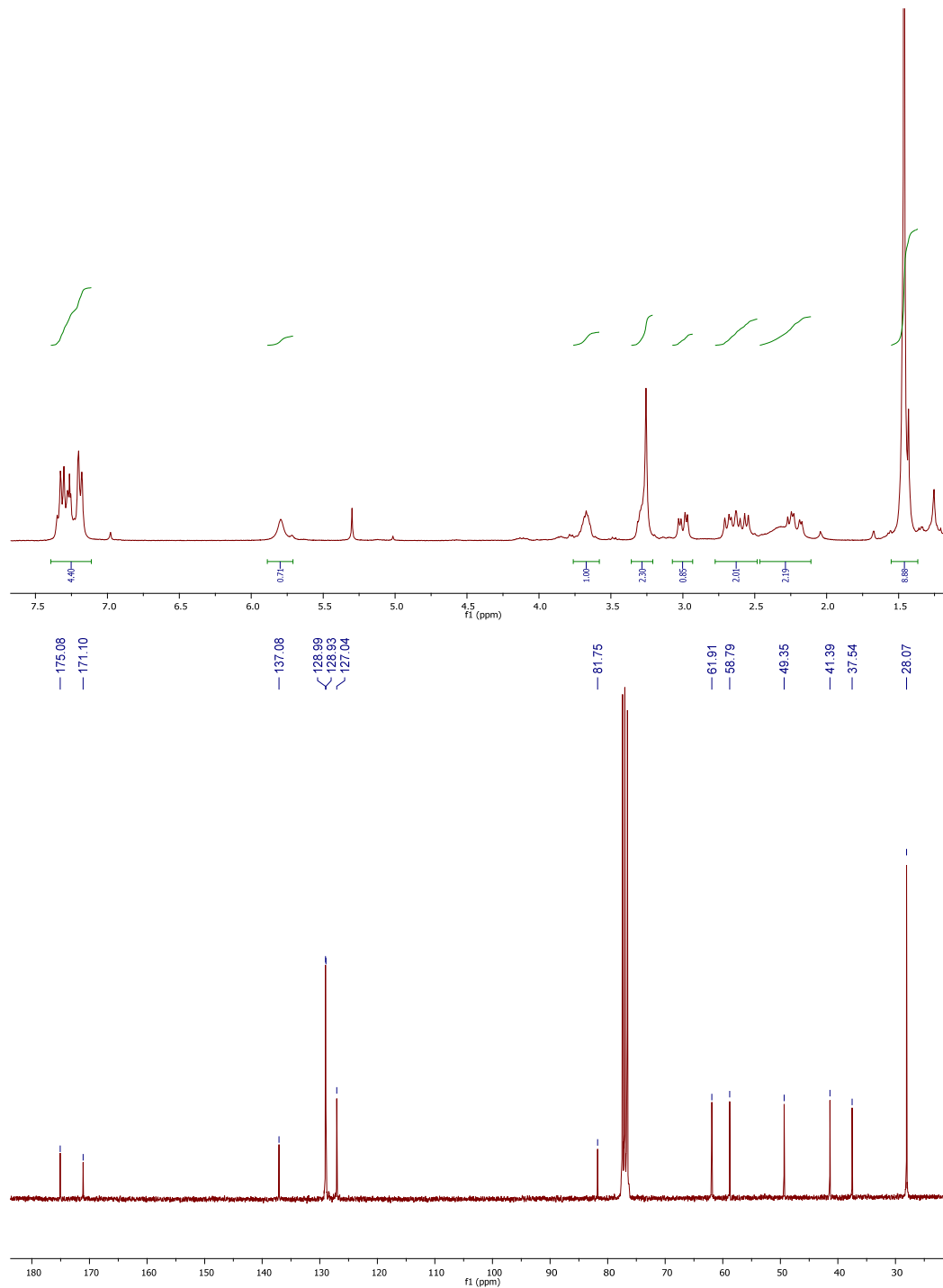

Supplement: Figure S8 — NMR Spectra for new compounds 2–8 (PDF) [file pone.0053231.s008.pdf]
