# Supplementary material for: Highly Functionalized 1,2–Diamino Compounds through Reductive Amination of Amino Acid-Derived β–Keto Esters
Source: PLoS One. 2013 Jan 7;8(1):e53231. doi: 10.1371/journal.pone.0053231 (PMC3538761; doi:10.1371/journal.pone.0053231)
Supplement: Figure S6 — X-Ray packing of compound 7a. (PDF) [file pone.0053231.s006.pdf]

Figure S6. X-Ray structure of (a) the *pseudo*-centrosymmetric dimer, (b) one chain, (c) one layer and (d) packing along **a** axis for compound **7a**. Dashed lines indicate hydrogen bonds.

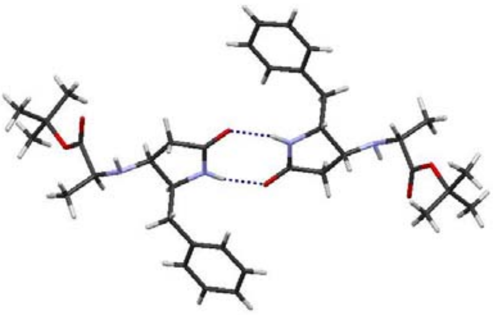

(a)

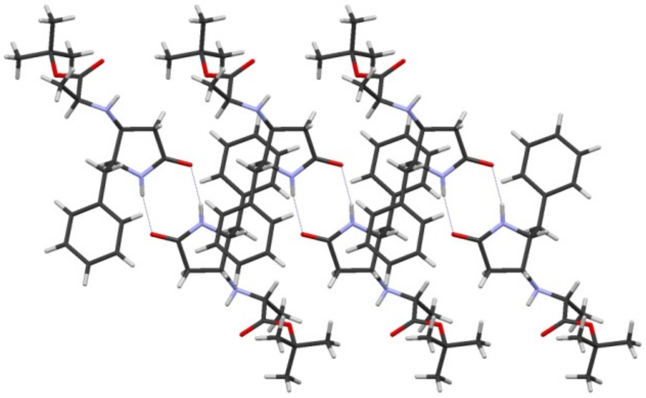

(b)

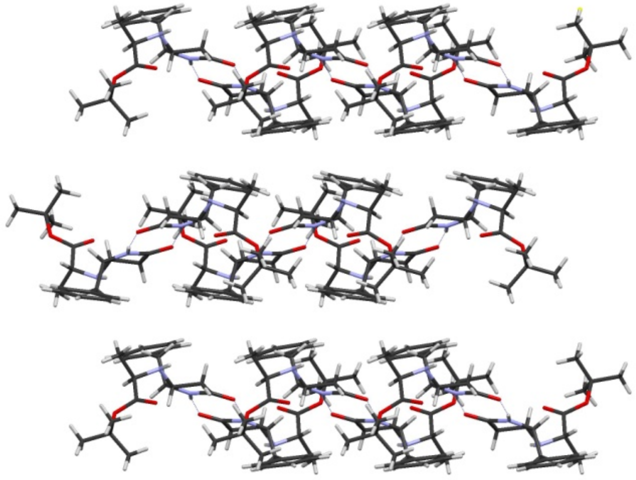

(c)

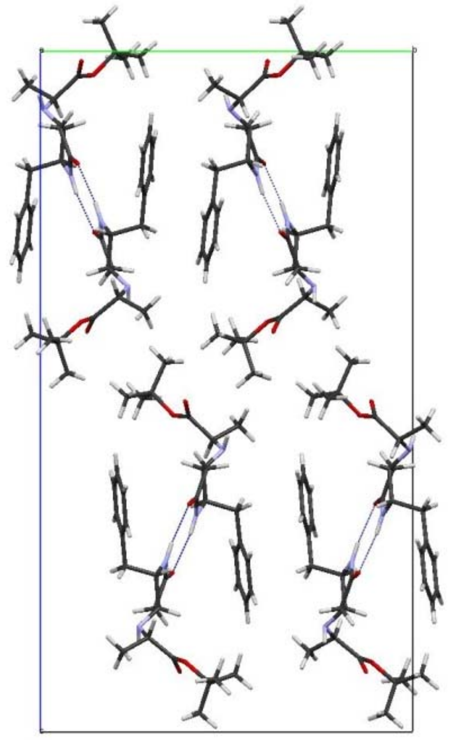

(d)
