# Supplementary material for: Highly Functionalized 1,2–Diamino Compounds through Reductive Amination of Amino Acid-Derived β–Keto Esters
Source: PLoS One. 2013 Jan 7;8(1):e53231. doi: 10.1371/journal.pone.0053231 (PMC3538761; doi:10.1371/journal.pone.0053231)
Supplement: Figure S7 — X-Ray packing of compound 7b. (PDF) [file pone.0053231.s007.pdf]

Figure S7. X-Ray structure of (a) the centrosymmetric dimer, (b) one chain, (c) the sheet and (d) packing along **c** axis for compound **7b**. Dashed lines indicate intermolecular contacts.

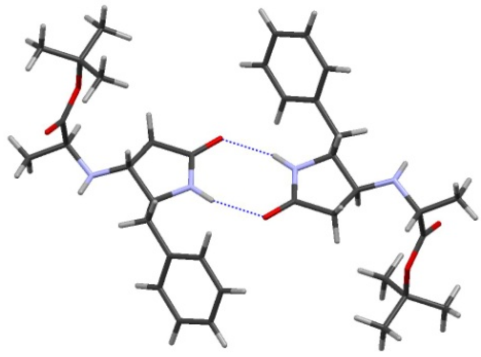

(a)

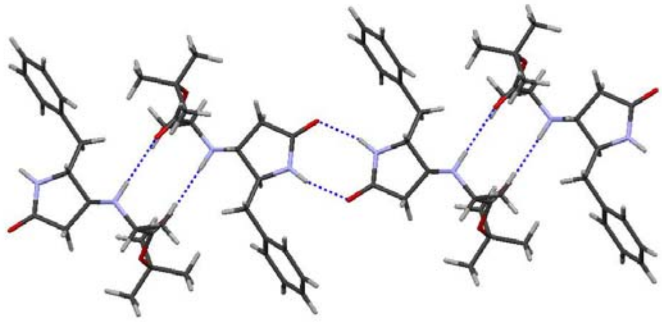

(b)

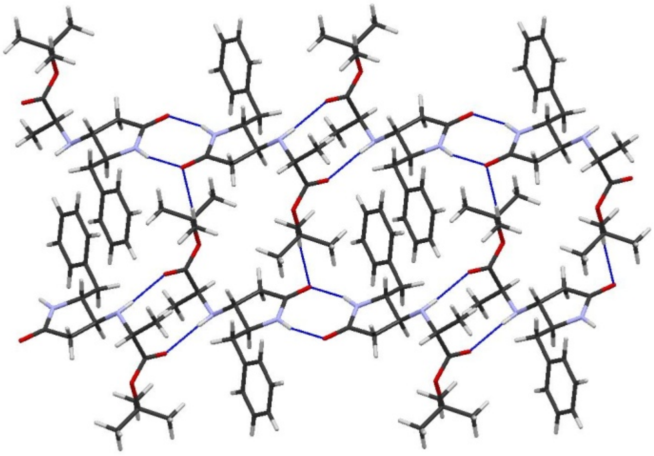

(c)

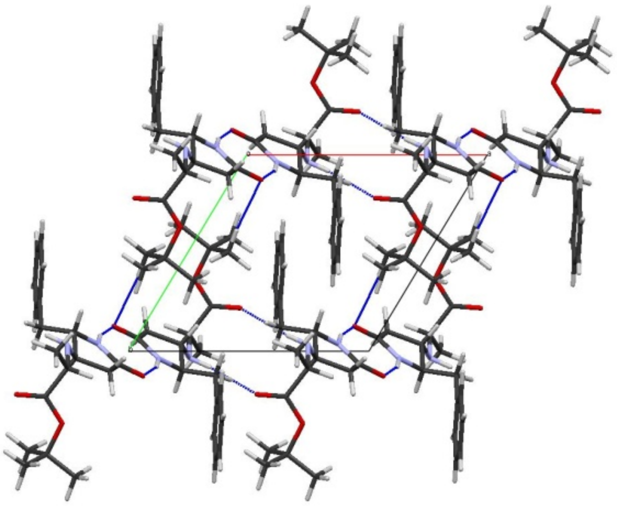

(d)
