# Supplementary material for: Highly Functionalized 1,2–Diamino Compounds through Reductive Amination of Amino Acid-Derived β–Keto Esters
Source: PLoS One. 2013 Jan 7;8(1):e53231. doi: 10.1371/journal.pone.0053231 (PMC3538761; doi:10.1371/journal.pone.0053231)
Supplement: Table S1 — Direct reductive amination trials. (PDF) [file pone.0053231.s009.pdf]

Table S1. Direct reductive amination trials

Entry	Aditive[a] (Solvent) Temp.[b]	[H]	Time (days)	HPLC peaks area (%)[c]						
				**2a**	**2b**	**3a**	**3b**	**3c**	Interm.	Starting **1**
1	$Ti(O^iPr)_4$ (CH_2Cl_2)	$NaBH_3CN$	1	8.9	10.6	–	–	–	–	79.0
2	AcOH (CH_2Cl_2)	$NaBH(OAc)_3$	2	8.1	15.8	–	–	–	–	71.8
3	AcOH ($CHCl_3$)	$NaBH_3CN$	1	39.0	53.1	–	–	–	–	–
4	AcOH (MeOH) 50 ºC	$NaBH_3CN$	3	30.1	34.2	4.7	7.6	6.2	–	–

[a] $Ti(O^iPr)_4$ (2 equiv); AcOH (0.5 equiv). [b] Room temperature, unless otherwise indicated. [c] Column:X-Bridge (0.21 x 10 cm, 3.5 μm). Eluent: Gradient: 20-100% H_20 (0.1 HCO_2H)/ ACN (0.08% HCO_2H), 15 min., 0.5 mL/min.
