# Supplementary material for: Highly Functionalized 1,2–Diamino Compounds through Reductive Amination of Amino Acid-Derived β–Keto Esters
Source: PLoS One. 2013 Jan 7;8(1):e53231. doi: 10.1371/journal.pone.0053231 (PMC3538761; doi:10.1371/journal.pone.0053231)
Supplement: Table S2 — Indirect reductive amination experiments. (PDF) [file pone.0053231.s010.pdf]

Table S2. Indirect reductive amination experiments

Entry	Aditive[a] (solvent) Temp. [b]	Time (days)	[H]	Time	HPLC peaks area (%)[c]						
					**2a**	**2a**	**3a**	**3b**	**3c**	Interm.	Starting **1**
1	$Ti(O^iPr)_4$ (MeOH)	1	$NaBH_4$	1	19.5	34.4	–	–	–	6.0	17.9
2	$Ti(O^iPr)_4$ (MeOH)	1	$NaBH_3CN$	1	11.5	15.8	–	–	–	7.8	53.8
3	$Ti(O^iPr)_4$ (MeOH)	1	$NaBH(OAc)_3$	1	0.9	1.8	–	–	–	39.5	52.3
4	$Ti(O^iPr)_4$ (DCE)	4	$NaBH(OAc)_3$	7	13.8	31.4	–	–	–	–	47.3
5	$Ti(O^iPr)_4$ (DCE)	4	$NaBH(OAc)_3$ 50ºC	3	12.8	36.9	–	–	–	–	35.6
6	$Ti(O^iPr)_4$ I_2 (DCE)	4	$NaBH(OAc)_3$ 50ºC	3	8.5	25.6	–	–	–	–	59.3
7	$Ti(O^iPr)_4$ (DCE)	4	$NaBH_3CN$	3	31.0	36.7	–	–	–	–	1.2
8	$Ti(O^iPr)_4$ no solvent 50ºC	0,3	$NaBH_3CN$ MeOH	3	14.0	16.4	–	–	–	3.5	57.3
9	$Ti(O^iPr)_4$ (CH_2Cl_2)	1	$NaBH(OAc)_3$ $NaBH_3CN$	1	28.2	48.9	7.2	5.4		–	–
10	AcOH 0.5 (CHCl3)	4	$NaBH_3CN$	3	13.2	24.0	10.0	29.0		–	–
11	AcOH 0.5 (CHCl3) 50ºC	4	$NaBH_3CN$	3	2.7	4.7	19.6	45.8		–	–
12	AcOH 0.5 (MeOH, 50ºC)	4	$NaBH_3CN$	3	25.6	25.6	6.3	19	7.9	–	–
13	AcOH 1.0 (CHCl3)	6	$NaBH_3CN$	1	14.2	26.3	13.3	25.8		–	–
14	AcOH 1.0 (CHCL3 50ºC)	1	$NaBH_3CN$	4	13.6	35.6	13.7	13.6	18.9	1.4	–
15	AcOH 1.0 (CHCL3)	4	$NaBH_3CN$	4	0.5	0.6	18.4	33.9	30.9	–	–
16	AcOH 1.0 (CHCL3, 50ºC)	2	$NaBH_3CN$	4	0.6	0.8	21.3	26.9	36.4	–	–
17	AcOH 1.0	6	H_2/Pd/C	1	–	–	–	–	–	24.0	68.4

	(MeOH)										
18	CAN 5% (MeOH)	3	$NaBH_3CN$	0.5	1	1.5	–	–	–	30.0	50.1
19	CAN 5% (MeCN)	3	$NaBH_3CN$	1	18.3	20.0	–	–	–	–	51.0
20	$LaCl_3$ 10% (CH_2Cl_2)	6	$NaBH_3CN$ MeOH	1	9.1	21.3	4.0	4.9	5.2	10.1	32.6
21	$ZnCl_2$ 1.0 (MeOH)	1	$NaBH_3CN$	1	16.0	15.9	6.0	6.4	8.5	2.8	46.0

[a] Ti(O^{i}Pr)$_4$ (2 equiv); AcOH (0.5 equiv). [b] Room temperature, unless otherwise indicated. [c] Column:X-Bridge (0.21 x 10 cm, 3.5 µm). Eluent: Gradient: 20-100% H_20 (0.1 HCO_2H)/ ACN (0.08% HCO_2H), 15 min., 0.5 mL/min.

Intermediates = enamines (E+Z isomers).
